# Italian Food? Sounds Good! Made in Italy and Italian Sounding Effects on Food Products' Assessment by Consumers

**DOI:** 10.3389/fpsyg.2021.581492

**Published:** 2021-03-03

**Authors:** Flavia Bonaiuto, Stefano De Dominicis, Uberta Ganucci Cancellieri, William D. Crano, Jianhong Ma, Marino Bonaiuto

**Affiliations:** ^1^Facoltà di Economia, Universitas Mercatorum, Rome, Italy; ^2^Dipartimento di Medicina Sperimentale, Sapienza Università di Roma, Rome, Italy; ^3^Department of Nutrition, Exercise and Sports, University of Copenhagen, Copenhagen, Denmark; ^4^CIRPA—Centro Interuniversitario di Ricerca in Psicologia Ambientale, Sapienza Università di Roma, Rome, Italy; ^5^Dipartimento di Scienze Della Società e della Formazione D'Area Mediterranea, Università per Stranieri “Dante Alighieri” di Reggio Calabria, Reggio Calabria, Italy; ^6^School of Social Science, Policy & Evaluation, Claremont Graduate University, Claremont, CA, United States; ^7^School of Psychology and Behavioral Sciences, Zhejiang University, Hangzhou, China; ^8^Dipartimento di Psicologia dei Processi di Sviluppo e Socializzazione, Sapienza Università di Roma, Rome, Italy

**Keywords:** food, Italian sounding, made in Italy, reputation, willingness to pay (WTP)

## Abstract

Italian Sounding—i. e., the Italian appearance of a product or service brand irrespective of its country of origin—represents a global market phenomenon affecting a wide range of economic sectors, particularly the agro-food sector. Although its economic impact has been repeatedly stressed from different points of view (policy, economy, culture, etc.), systematic scientific knowledge regarding its social–psychological bases is lacking. Three studies carried out in three different countries (Italy, China, and USA) address this literature gap. Different consumer groups (both native and/or non-native) are targeted regarding major product categories pre-selected categories, which are the major Italian food goods within the specific country according to piloting (oil and/or pasta). In each study, the main independent variable (product version) has been manipulated by presenting real product images (previously pre-selected within the tested food category in each country market), whose “Italianness” degree is effectively manipulated by the main study variable (product version) across three or four levels (Protected Designation of Origin Made in Italy, Made in Italy, Italian Sounding, and Generic Foreign). Main hypotheses are tested via a survey with the specific product images administered to samples in Italy (*N* = 204, 148 Italians and 56 non-Italians), China (*N* = 191, 100 Chinese and 91 non-Italian expatriates in China), and the USA (*N* = 237 US citizens). Across the three studies, results show that Made in Italy products, compared to the other ones, are advantaged in terms of the main dependent variables: reputation profile, general reputation, attitude, and willingness to pay (WTP). Moreover, Italian Sounding products are endowed with corresponding significant advantages when compared to the Generic Foreign by non-Italian samples (although to a different degree according to the different sub-samples). Results reveal the specific social–psychological profile of Italian Sounding products in terms of either weaknesses or strengths when compared to both Made in Italy products and Generic Foreign ones, differently in the eyes of Italian and non-Italian consumers across different countries. Finally, consistently across the three studies, the extent to which a food product is perceived to be Italian increases consumers' WTP for that product, and this effect is consistently mediated by the product's reputation.

## Introduction

The food industry is the second most important sector of Italian economy, making Italy the 10th exporter of this sector in the world [ISMEA (Istituto di Servizi per il Mercato Agricolo Alimentare), 2017]. Agro-food “Made in Italy” products—with features evoking an “Italian” concept in the world, including history, culture, and tradition (Napolitano et al., 2015; Temperini et al., 2016)—are typical goods of the Mediterranean diet [Antimiani and Henke, 2007; ISMEA (Istituto di Servizi per il Mercato Agricolo Alimentare) and Fondazione Qualivita, 2018], and they currently spearhead Italian exports in terms of technologies, procedures, and intrinsic transformation of raw materials (Carbone and Henke, 2012; Caiazza and Volpe, 2014; Coldiretti, 2015). The European Commission has adopted several regulations on the application of EU quality schemes for the agro-food sector (Barjolle and Sylvander, 2000; European Commission, 2016), in order to protect typical products and to provide quality guarantee (Van der Meulen, 2015). The compulsory affixing of PDO, Protected Designation of Origin; PGI, Protected Geographical Indication; TSG, Traditional Specialty Guaranteed labels to products ensures consumers' safety with certification of working methods, reputation of places of production, traceability, and risk management of food (World Trade Organization, 1994). Italian agro-food distinctive products constitute about 80% of domestic exports in the food sector, with a recent growing appreciation especially in China (Huliyeti et al., 2008; Snaiderbaur, 2009; Vianelli et al., 2012a).

### The Italian Sounding Phenomenon

Because of its worldwide known high-quality standards, the Italian agro-food market is currently facing various food counterfeiting (or similar) phenomena (Nicoletti et al., 2007; Iaricci et al., 2010; Montanari et al., 2016), especially the increasingly widespread phenomenon of the so-called “Italian Sounding” (IS), which consists in proposing to consumers products whose name, image, shape, and place of production are associated with “typically Italian” features. In IS, either the product name may recall the “original” one, as the American “Parmesan” cheese, or the brand may be invented, although “sounding” Italian as the “Da Vinci” or “Gattuso” tomato sauces. Colors evoking the Italian flag and images of famous Italian landscapes or monuments—e.g., the gulf of Naples, the tower of Pisa—reproduced on the label and packaging are other frequently used strategies [OECD (Organization for Economic Cooperation Development), 2008; Canali, 2012; Carreño and Vergano, 2016; Federalimentare, 2016].

This contemporary phenomenon can be partly framed within the very well-known Country of Origin (COO) effect, namely, the process by which “country of origin has a considerable influence on the quality perceptions of a product” (Bilkey and Ness, 1982, p. 89; see also Mainolfi, 2010; Marino and Mainolfi, 2013). IS is however different, as it is based on exploiting an alleged COO, on the basis of an ambiguity of the product's origin. Quite often, the IS product officially declares its “real” country of origin (i.e., via a correct labeling “made in”), while at the same time presenting an Italian “allure” endowed by means of some of its peripheral cues (e.g., stereotypical colors or images, as well as a name which “sounds” Italian, etc.). This communication and marketing compromise solution is therefore not counterfeiting its “true” country of origin, but at the same time it mimics some features of another “different” country of origin (specifically, Italy) or of its most classical and typical food products or culinary recipes. The informational cues that are crucial to convey an alleged COO (parallel to the “made in” one) can be of different kinds, and they are used as a basis to evaluate the product, by different kinds of stakeholders including consumers from different geographical areas too (e.g., Bilkey and Ness, 1982). Though this is mostly a social-cognitive process, it is framed within broader social systems, ending up in social–psychological implications for consumers' social inferences and attributions, as well as consumption decision-making processes, up to less or more stable consumer habits (fads or trends), and situated social identities in terms of a consumer's practices and communities (e.g., Busacca et al., 2006; Boatto et al., 2016).

More specifically, IS is based on the Country Sound Branding (CSB) construct, i.e., the construction of a brand recalling a non-real Country of Origin Image (COI), a variable often affecting consumers' attitude toward products (Erickson et al., 1984; Roth and Romeo, 1992; Phau and Prendergast, 2000; Rosenbloom and Haefner, 2009; Samiee, 2010; Bertoli and Resciniti, 2012; Aichner, 2013). Companies may use a strongly positive COI in order to increase their products' attractiveness (Usunier, 2006, 2011; Bursi et al., 2012; Vianelli et al., 2012b), also taking advantage of the fact that information on the product's origin may not be immediately accessible for many brands (Zhou et al., 2010).

### Consumers' Cultural Differences

As already mentioned above (Bilkey and Ness, 1982), since its beginning, the COO literature has been aware of the fact that the COO effect depends on the stakeholders' point of view, first of all regarding consumers' different geographical and cultural areas. The subtlety of the interplay among the official declaration of the true country of origin (a non-Italian country “made in”), on one side, and of the alleged country of origin suggested or evoked by some packaging peripheral features (Italian-like colors or images or wording), on the other side, is not perceived, and/or cognitively or affectively treated in the same way from each consumer. Of course, a consumer's specific knowledge and past experience of the specific country and with its products can come into play here, as well as other factors related either to a consumer's attention process and/or to her/his personal, social, and place identity, regarding cultural dimensions too. These factors can either facilitate or hinder psychological dynamics, and particularly social-cognitive ones, at the basis of the IS phenomenon. In fact, the CSB uses the favorable misclassification strategy, so that the brand is perceived as coming from a country with a more positive image or reputation, at least in that very specific sector, than that of the real COO (Balabanis and Diamantopoulos, 2011), in the eye of that consumer. Weaker and not well-known brands especially benefit from this strategy (Ahmed and D'Astous, 1996; Ahmed et al., 2002). Recently, the ability of an accurate perception of the brand's COI by customers has been questioned (Liefeld, 2004; Magnusson et al., 2011; Checchinato et al., 2013), a detail being generally communicated by the seller rather than required by consumers. However, consumers do associate the brand to a specific country, thus affecting the overall final image, or reputation, and attitude toward the brand itself (Liefeld, 2004; Balabanis and Diamantopoulos, 2008, 2011). This intentional ambiguity of the brand name, being printed on the product label, is very widespread as it is much more visible than the label of origin (the official “made in”), thus requiring a lower learning effort from the consumer (Thakor, 1996; Thakor and Lavack, 2003). IS products thus contribute to uncertainty and confusion, generating doubts, or false certainties, in consumers on the actual origin of Italian products. Possible negative consequences of the growing phenomenon of IS are delocalization of production, choice of non-local raw materials, and loss of quality, possibly causing the complete disappearance of authentic “Made in Italy” products (EURISPES, 2013). Such a complex scenario—where product information regarding its (real and/or alleged) country of origin interplay with the consumer's cultural background within a certain national market interconnected with the global one—thus requires deepening the investigation of psychological and social-cognitive dynamics connected to the IS phenomenon within a given national market according to different sub-samples existing in such a context. Studies did not address this issue systematically, and this is why, in two of the three studies presented here, two different sub-samples are considered and their results contrasted in order to test how the IS effect depends not only on the interplay among a specific market and the perceived Italianness of the food product but also on the specific perspective of the local stakeholder: a certain consumer group vs. another one within the same geographical and cultural area, considering both the West and the East, although all located within one only scenario: the contemporary global market.

### Food Reputation

Over the last decades, the advent of the Internet, new media, social networks, and online communities have gradually assimilated the world to a “global village,” where the mediation of experiences via multiple social actors is causing a major return of the construct of reputation (Bonaiuto et al., 2012), especially in large electronic markets, such as eBay and Amazon (Ulgado, 2002; Kuwabara, 2005; Chang et al., 2006; Utz et al., 2012), where the lack of physical interaction with sellers and goods calls for a greater need for trust, compared to traditional markets.

Based on perception (Zajonc, 1980; Isen, 1984; Lerner and Keltner, 2000; Winkielman and Cacioppo, 2001; Herzog and Stark, 2004; Graziano, 2010) and influenced by advertising (Babiloni et al., 2007; Graziano, 2010), the construct of reputation (Emler, 1990; Conte and Paolucci, 2002; Marmo, 2007; Mutti, 2007) can be defined as believed effects that any social agent (ranging from a person to a company up to a country) can have (Emler, 1990; Bromley, 1993, 2001; Palmonari et al., 2002; Bonaiuto et al., 2012).

In an organizational context, a company's reputation affects the relationship between quality and price (Klein and Leffler, 1981; Shapiro, 1983; Allen, 1984; Gorton, 1996; Winfree and McCluskey, 2005; van Riel and Fombrun, 2007). At the time of purchase, consumers generally observe intrinsic (e.g., freshness, flavor) and extrinsic (e.g., price, label) quality signals more than quality attributes (Steenkamp, 1990). A study on the evaluation of Bordeaux wine shows that the premium price associated with better individual and collective (or group) reputation far exceeds that associated with improvements in current quality (Landon and Smith, 1998).

In this context, food reputation is becoming a particularly stimulating research field, as foods and drinks affect the life of an individual on a physical, psychological, and social level, and are involved in many other social problems, such as globalization (e.g., the so-called “McDonaldization” phenomenon; Ritzer, 1996) or the exploitation of natural resources (Kuisel, 1991; Zimmet, 2000; Leatherman and Goodman, 2005; U.S. Department of Agriculture and U.S. Department of Health and Human Services, 2011). Foods and drinks, and the whole systems involving them and revolving around them, are the core of a renewed appreciation of the quality issue, e.g., according to three fundamental features within the Slow Food paradigm: good, clean, and fair (Petrini, 2005, 2010). Therefore, within such a contemporary complex global scenario, COO became particularly relevant within the agro-food sector, where it contributes to a certain food item assessment in terms of interest toward its origin, which promotes its positive image evaluation and consequently its consumption (Yeh et al., 2010): moreover, COO can interplay with other food features, but not many studies have addressed the interplay among COO, on the one hand, and other food features, on the other hand (e.g., Loureiro and Umberger, 2007). Finally, geographical and cultural differences across consumers' sub-samples can become relevant here too.

Within the social–psychological literature, food features have been traditionally investigated according to some relevant dimensions affecting consumers' choices (Magnusson et al., 2003) and via measuring scales such as the *Reasons for Eating Scale* (Harmatz and Kerr, 1981), the *Food Choice Questionnaire* (Steptoe et al., 1995), and broader conceptual frames on food features (Conner and Armitage, 2002; Olivero and Russo, 2008). The *Food Reputation Map* (FRM) is probably the first validated set of scales which specifically and explicitly targets the reputation of food products by encompassing the wider array of their features: in fact, FRM includes six main areas of food reputation, articulated into 23 dimensions determining food attractiveness, based on past experiences and future expectations; moreover, such a paradigm has also been already deployed in terms of geographical and cultural differences, by testing it with consumers from both a Western and an Eastern area (Bonaiuto et al., 2012, 2017; De Dominicis et al., 2020). It is therefore the most updated and complete paradigm to tackle the issue of a food product's features, and it can easily be adopted to study and test issues related to the COO effect paradigm, such as the IS phenomenon seems (partly, at least) to be, considering different consumer cultural sub-samples within the same geographical area. A product's geographical and cultural origin perception and the consequent food feature assessment being dependent on the COO effect would of course affect the consumer's attitude toward it as well as her/his final decision-making in terms of food product purchasing, thus affecting that consumer's willingness to pay (WTP) a certain amount of money for that food item.

### Willingness to Pay

Consumers' food choices concern physical characteristics of the products themselves and psychological factors (Rozin et al., 1986), including the perception of the risk to food safety (Yeung and Morris, 2001; De Jonge et al., 2004). On top of that, in the last decades, the agro-food market has recorded an increased demand for organic, natural, and local products (Thompson, 1998; Dimitri and Greene, 2002), often motivated by a growing concern for health (Huang, 1996; Makatouni, 2002; Honkanen et al., 2006) and by the perception of these products being more environmentally friendly and more favorable for small-scale agriculture and for local rural communities (Underhill and Figueroa, 1996; Williams and Hammit, 2000, 2001): as a consequence, WTP the premium prices generally required for high-quality products increases (Suryanta, 2000; Loureiro and Hine, 2002; Wang and Sun, 2003; Batte et al., 2007). For example, consumers were found willing to pay a premium price for fresh national meat products with a PGI label in Spain (Loureiro and McCluskey, 2000) and supported compulsory labeling policies (Schupp and Gillespie, 2001), often with the WTP a high premium (Loureiro and Umberger, 2007). However, in other cases, a reduction in the price of the product with uncontrolled origin was sufficient for the consumer to be indifferent between the two (Unterschultz et al., 1997).

The WTP premium prices for quality products has been found higher among families with children (Thompson and Kidwell, 1998) or with few members (Loureiro and Hine, 2002), families with high income (Wang and Sun, 2003), and women (Loureiro and Umberger, 2007). WTP has been measured mainly via the *Contingent Valuation* (CV) method (Hanemann, 1984).

Studies on reputation and WTP applied to agro-food products are still limited. It is particularly needed to understand how the “made in” (here specifically, “Made in Italy”) products are perceived and how much people are willing to pay for them, in order to contrast the IS (as a case of CSB) phenomenon, increasingly spreading in many countries, which has a negative impact on the Italian market (Canali, 2012; Carreño and Vergano, 2016; Federalimentare, 2016). Moreover, while such a literature often advocated that the IS phenomenon has a negative economic impact on the global Made in Italy agro-food sector, with its financial impact considered at a macro-economic level—recent estimates from Italian institutional bodies or economic specialized press placed it in the range of €50–90 billion per year and, in some cases, near to almost €100 billion per year—there are not many evidences of how the IS phenomenon affects the single individual's micro-economic decision (e.g., in terms of WTP for purchasing a single specific agro-food item by the single consumer). IS spreading is most prevalent in China and the USA: according to some sources, most counterfeit products on the European market come from China (Cheung and Prendergast, 2006; Lin, 2011; Zimmerman, 2013), and the US market is the one with the largest amount of false Italian food (IPR Desk NY, 2010, 2011; Federalimentare, 2016), although both countries register an increasing level of appreciation for Italian products (Girardelli, 2004; Huliyeti et al., 2008; Vianelli and Pegan, 2014). As for the USA, IS is particularly frequent in metropolitan areas, with large Italian-American communities and above-average incomes, and it results from the need for US companies—often created by Italian-Americans and subsequently absorbed by multinationals—to respond to the increasing demand for Italian food (Vianelli and Marzano, 2013).

Given the paucity of studies addressing this set of phenomena, and particularly the lack of studies on the social–psychological processes regulating IS consumers' choices, the present research is developed with the aim of investigating the IS effects on consumers' assessment of agro-food products associated to Italy, and their consumption choices for such targets. The central question of the research is to determine whether, for a food product, the COO label, in terms of IS, can influence first of all its “Italianness,” then its general reputation and related specific reputation features, and, consequently, consumers' attitude toward that product, up to her/his WTP for that food item, by comparing different forms of products associated to Italy (*Made in Italy*; *IS*; *Generic Foreign*), within different cultural contexts and consumers sub-samples.

Three studies were designed to test this general aim. The first was conducted to compare the perception of two products typically associated with Italy (oil and pasta) by Italian and non-Italian subjects within the EU and to measure the effects of the product label on perception, attitude, reputation, and WTP for that product (typically associated to Italy), by using four different product forms (*PDO Made in Italy, Made in Italy, IS*, and *Generic Foreign*).

The second study was performed to measure the effects of the product label on perception, attitude, reputation, and WTP for a product (typically associated to Italy), by using three or four different product forms (*PDO Made in Italy, Made in Italy, IS*, and *Generic Foreign*) and by assessing more detailed reputational profiles by means of a standard tool (Bonaiuto et al., 2017; De Dominicis et al., 2020) in China considering two different sub-samples (Chinese and non-Chinese), which are relevant for their different cultural background in that national market (Huliyeti et al., 2008; Vianelli and Pegan, 2014). The third study was performed to measure the effects of the product label on perception, attitude, reputation, and WTP for a product (typically associated to Italy), by using three different product forms (*Made in Italy, IS*, and *Generic Foreign*) and by assessing detailed reputational profiles by means of a standard tool (Bonaiuto et al., 2017; De Dominicis et al., 2020) in the USA (Cembalo et al., 2008).

## Study 1

### Aim and Hypotheses

The main aim of Study 1 is to investigate how two sub-samples (Italian vs. non-Italian within the EU) in an Italian and EU context (respectively) perceive (in terms of reputation and attitude) and are willing to pay for two agro-food products typically associated to Italy (oil and pasta), presented in four forms, differentiated by label: *PDO Made in Italy, Made in Italy, Italian Sounding*, and *Generic Foreign*.

It is thus expected that^1^:

*H1:* The product form or label has an effect on reputation. The reputation is more positive for the Italian products (particularly PDO) compared to the *Italian Sounding* products, and this one compared to the *Generic Foreign*; further significant differences can emerge between Italian and non-Italian EU sub-samples.
*H2:* The product has an effect on attitude. In particular, the attitude is more positive for the Italian products (particularly PDO) compared to *Italian Sounding* products, and this one compared to the *Generic Foreign*; further significant differences can emerge between Italian and non-Italian EU sub-samples.
*H3:* The product has an effect on WTP. The WTP is higher for the Italian products (particularly PDO) compared to *Italian Sounding* products, and this one compared to the *Generic Foreign*; further significant differences can emerge between Italian and non-Italian EU sub-samples.

### Method

#### Participants, Procedure, and Materials

The survey reached a total of 204 subjects (*M* = 97; *F* = 107). A total of 148 were Italians living in Italy, 52% women, average age 33.3 (*SD* = 13.6); 56 were non-Italian, European Union citizens (23.2% UK, 16.1% Germany, 16.1% Spain, 7.1% Croatia, 7.1% France, and 30.4% other EU country), 53.6% women, average age 26.5 years (*SD* = 8.7). Electronic data collection was performed in January 2014 via an online survey in two versions (Italian and English), using various social media platforms (e.g., Facebook, Twitter). Each subject randomly received the questionnaire (Italian/English), concerning only one of the two selected food products (olive oil or pasta), and was asked to fill in the questionnaire observing the images of the four different products reported in it, presented in the following order: *PDO Made in Italy, Made in Italy, Italian Sounding*, and *Generic Foreign*.

In order to identify the products to be investigated, a pre-test was conducted, asking subjects to indicate some of the best known and most consumed Italian foods, and measuring the subjects' perception of different products' “Italianness” and origin. Pre-test subjects were firstly asked to list six food products that came to their mind when thinking of Italian food and subsequently to list six Italian food products they consumed most. On the basis of pre-test results, pasta and olive oil were selected. The different actual products were then selected on the basis of those actually on sale in Italian supermarkets at the time (avoiding most renowned brands in order to escape from potential strong familiarity effects).

The four selected olive oil products were as follows: “Garum olio extravergine di oliva D.O.P. Colline salernitane” as *PDO Made in Italy*, “Olio del Fraticello olio extravergine di oliva” as *Made in Italy*, “Fígaro extra virgin olive oil” as *Italian Sounding*, and “Natives olivenöl extra” as *Generic Foreign* (see Appendix A).

The four selected pasta products were as follows: “Spaghetti Gentile Pasta di Gragnano I.G.P.” as *PDO Made in Italy*, “Spaghetti Pasta Zara” as *Made in Italy*, “Spaghetti Milaneza” as *Italian Sounding*, and “Spaghetti Riesa Hartweizennudeln” as *Generic Foreign* (see Appendix A).

#### Measures

The questionnaire was produced in two similar versions, differentiated by product type (pasta or olive oil), and administered in the research with a 2 × 4 design: 2 different samples (Italian vs. EU non-Italian) × 4 product forms (*PDO Made in Italy, Made in Italy, Italian Sounding*, and *Generic Foreign*). The questionnaire includes several scales, repeated for each of the four product forms (*PDO Made in Italy, Made in Italy, Italian Sounding*, and *Generic Foreign*), and a final section concerning socio-demographic data (gender, age, country of origin, and country of residence). The whole survey is available in the Supplementary Material of this article.

Three types of manipulation checks were run to measure the subjects' perception of the product's “Italianness” and origin. In the first (“Italianness intensity”), subjects were asked how Italian they thought the product was, on an 11-point Likert-type scale (from 0 “in no way” to 10 “completely”): “Secondo lei quanto è Italiano il prodotto di riferimento?” or “In your opinion, how much Italian is the product?” In the second (“Italianness probability”), they were asked how likely it was that the product was produced in Italy, on a five-point Likert-type scale, ranging from 0% (= “definitely produced abroad”) to 100% (= “definitely produced in Italy”), with +25% cumulative increasing steps: “Secondo lei quanto è probabile che il prodotto di riferimento sia prodotto in Italia?” or “In your opinion, how much is it likely that the product is produced in Italy?” In the third (“Italianness origin”), subjects were asked where they thought the product came from, and to answer either 0 “Abroad” or 1 “Italy”: “Secondo lei da dove proviene il prodotto?” or “In your opinion, where does the product come from?”

Ten seven-point evaluative semantic differential scales were used to measure the subjects' attitude toward the product: “Cattivo” vs. “Buono”; “Contraffatto” vs. “Autentico”; “Naturale” vs. “Artificiale”; “Genuino” vs. “Manipolato”; “Vero” vs. “Falso”; “Indesiderabile” vs. “Desiderabile”; “Senza certificato” vs. “Con certificato”; “Alta qualità” vs. “Bassa qualità”; “Alta fascia” vs. “Bassa fascia”; “Economico” vs. “Costoso” (in their English version as well, “Bad” vs. “Good”; “Counterfeit” vs. “Authentic”; “Natural” vs. “Artificial”; “Genuine” vs. “Manipulated”; “True” vs. “False”; “Undesirable” vs. “Desirable”; “Without certificate” vs. “With certificate”; “High quality” vs. “Low quality”; “High range” vs. “Low range”; “Economic” vs. “Expensive”). The rating scale was a Likert-type scale ranging from 1 (= “totally”) to 7 (= “totally”), with 4 (= “neither/nor”) being intermediate.

To measure the product's general reputation, one item was used, asking subjects to indicate the product's reputation on a seven-point Likert-type scale (from “completely negative” to “completely positive”): “Questa Pasta/Olio ha una reputazione” or “This Pasta/Oil reputation is.”

To measure WTP (Gil et al., 2000), subjects were asked how much they would be willing to pay for that product considering its average price, expressed in euros. Responses were given on an 11-point Likert-type scale, adapted from Hanemann (1984). WTP ranged from €2.50 to €7.50 for olive oil (with one step increase in the Likert scale corresponding to an increase of €0.50) and from €0.00 to €4.00 for pasta (with one step increase in the Likert scale corresponding to an increase of €0.40). The scale's middle point and range in euro was close to a possible national average price for the product in that period (€5.00 for oil, €2.00 for pasta). For the Italian sample, the “oil” item was: “Considerando che il costo medio al litro dell'olio extravergine di oliva è pari a circa €5.00, quanto sarebbe disposta/o a pagare se volesse acquistare un litro di PRODUCT NAME.” For the Italian sample, the “pasta” item was: “Considerando che il costo medio 500 g di pasta è pari a circa €2.00, quanto sarebbe disposta/o a pagare se volesse acquistare 500 g di PRODUCT NAME.” For the non-Italian EU sample, the “oil” item was: “Considering that the average price of a liter of extra virgin olive oil is about €5.00, how much would you be willing to pay if you would buy a liter of PRODUCT NAME.” For the non-Italian EU sample, the “pasta” item was: “Considering that the average price of 500 g of pasta is about €2.00, how much would you be willing to pay if you would buy 500 g of PRODUCT NAME.”

All statistical analyses were released using the SPSS version 27 software.

#### Manipulation Check

A manipulation check was performed to test whether the manipulation of the products, being PDO Made in Italy, Made in Italy, Italian Sounding, or Generic Foreign products, was effective in changing the perception of Italianness (intensity, probability, and origin) in our participants.

Two repeated-measures analyses of variance (ANOVAs) were performed to test the effects of product label on the dependent variable “Italianness intensity” (score 0–10) and “Italianness probability” (score 0–100%). The manipulation checks indicated an effect of the product label on Italianness intensity [*F*_(2.45, 496.25)_ = 657.4, *p* < 0.001, η*p*^2^ = 0.76] and on Italianness probability [*F*_(2.39, 464.9)_ = 579.43, *p* < 0.001, η*p*^2^ = 0.75], such that both dependent variables significantly decreased from PDO Made in Italy to Made in Italy to Italian Sounding to Generic Foreign products. Estimated marginal means comparisons showed significant differences across all four means (see values in Table 1) in each of the two dependent variables.

**Table 1:** Mean scores and *SD* of product label related to Italianness intensity, probability, and origin (Study 1).

**Product label**	**Italianness intensity**	**Italianness probability**	**Italianness origin**
	**M (SD)** **(*N* = 203)**	**M (SD)** ***N* = 195)**	**Italian%/foreign%** **(*N*)**
PDO made in Italy	8.54 (1.79)	4.33 (0.80)	97/3 (199)
Made in Italy	5.86 (2.38)	3.24 (0.99)	74.5/25.5 (192)
Italian sounding	1.62 (2.16)	1.64 (0.91)	10.3/89.7 (194)
Generic foreign	0.94 (1.73)	1.33 (0.68)	4.7/95.3 (193)

Furthermore, four binary logistic regressions were conducted to understand whether Italian origin (yes/no) was predicted by each product label. All four models were statistically significant (all *p* < 0.001) and predicted Italian (or non-Italian) origin as expected: the PDO Made in Italy and Made in Italy products were considered Italian products in 97 and 74% of cases, respectively, while the Italian Sounding and Generic Foreign products were considered foreign products in 89.7 and 95.3% of cases, respectively.

Therefore, it is possible to conclude that the manipulation was effective: the PDO Made in Italy product was perceived as more Italian than the Made in Italy product, which, in turn, was perceived as more Italian than the Italian Sounding product, which was finally perceived more Italian than the Generic Foreign product.

Finally, given its relevance for the present manuscript, we wanted to further corroborate the effect of the manipulation specifically for the Italian Sounding product. Therefore, we run an independent samples *t*-test comparing the perceived Italianness of the Italian Sounding product between the Italian and non-Italian samples. We expected that the Italian Sounding product should be perceived to be lower in Italianness by the Italian sample compared to the non-Italian sample. Results confirmed this expectation, showing that the Italian sample reported both lower Italianness intensity [*M* = 1.11, *SD* = 1.89, *t*_(201)_ = −6.02, *p* < 0.001] and Italianness probability [*M* = 1.44, *SD* = 0.74; *t*_(199)_ = −5.07; *p* < 0.001] than the non-Italian sample (*M* = 3.04; *SD* = 2.27; *M* = 2.21; *SD* = 1.07, respectively).

### Results

Cronbach's α was calculated to test the reliability of the attitude scale. Analyses show that the scale is reliable at all levels of measurement within the subjects (*PDO Made in Italy*, α = 0.90; *Made in Italy*, α = 0.88; *Italian Sounding*, α = 0.89; *Generic Foreign*, α = 0.92). In order to test H1, H2 and H3, a series of 2 (between-subjects factor: Italian vs. non-Italian) × 4 (within-subject factors: *PDO Made in Italy* vs. *Made in Italy* vs. *Italian Sounding* vs. *Generic Foreign*) mixed-model ANOVAs were run to verify the effect of the independent variables (nationality and product label) on the dependent variables (reputation, attitude, and WTP), for the two food products aggregated (preliminary analyses showed a general lack of significant differences on the main dependent variables among them). A series of protected *t*-test pairwise comparisons were also conducted in order to define significant differences between the individual levels of the two independent variables on the three dependent variables. Data analyses report the following results for the hypotheses, while all descriptive statistics are synthetized in Table 2.

**Table 2:** Mean scores and *SD* of product label related to attitude, reputation, and WTP (€) in each national sample (Italian vs. Non-Italian).

**Product label**	**Reputation (0–10)**		**Attitude (1–7)**		**WTP** **(€)**	
	**Italian** **M (SD)** **(*N* = 148)**	**Non-Italian** **M (SD)** **(*N* = 53)**	**Italian** **M (SD)** **(*N* = 138)**	**Non Italian** **M (SD)** **(*N* = 44)**	**Italian** **M (SD)** **(*N* = 147)**	**Non-Italian** **M (SD)** **(*N* = 53)**
PDO made in Italy	5.29 (1.06)	5.32 (0.85)	5.48 (1.13)	5.41 (0.90)	7.14 (2.30)	7.51 (1.73)
Made in Italy	3.87 (0.93)	4.30 (1.03)	3.73 (0.96)	4.44 (0.87)	4.13 (1.99)	5.30 (1.60)
Italian sounding	2.81 (1.17)	3.43 (1.17)	2.59 (1.04)	3.29 (0.96)	2.59 (1.65)	3.74 (2.10)
Generic foreign	3.17 (1.27)	3.92 (1.16)	3.08 (1.23)	3.97 (1.31)	2.96 (1.87)	5.09 (2.32)

**Figure 1:**
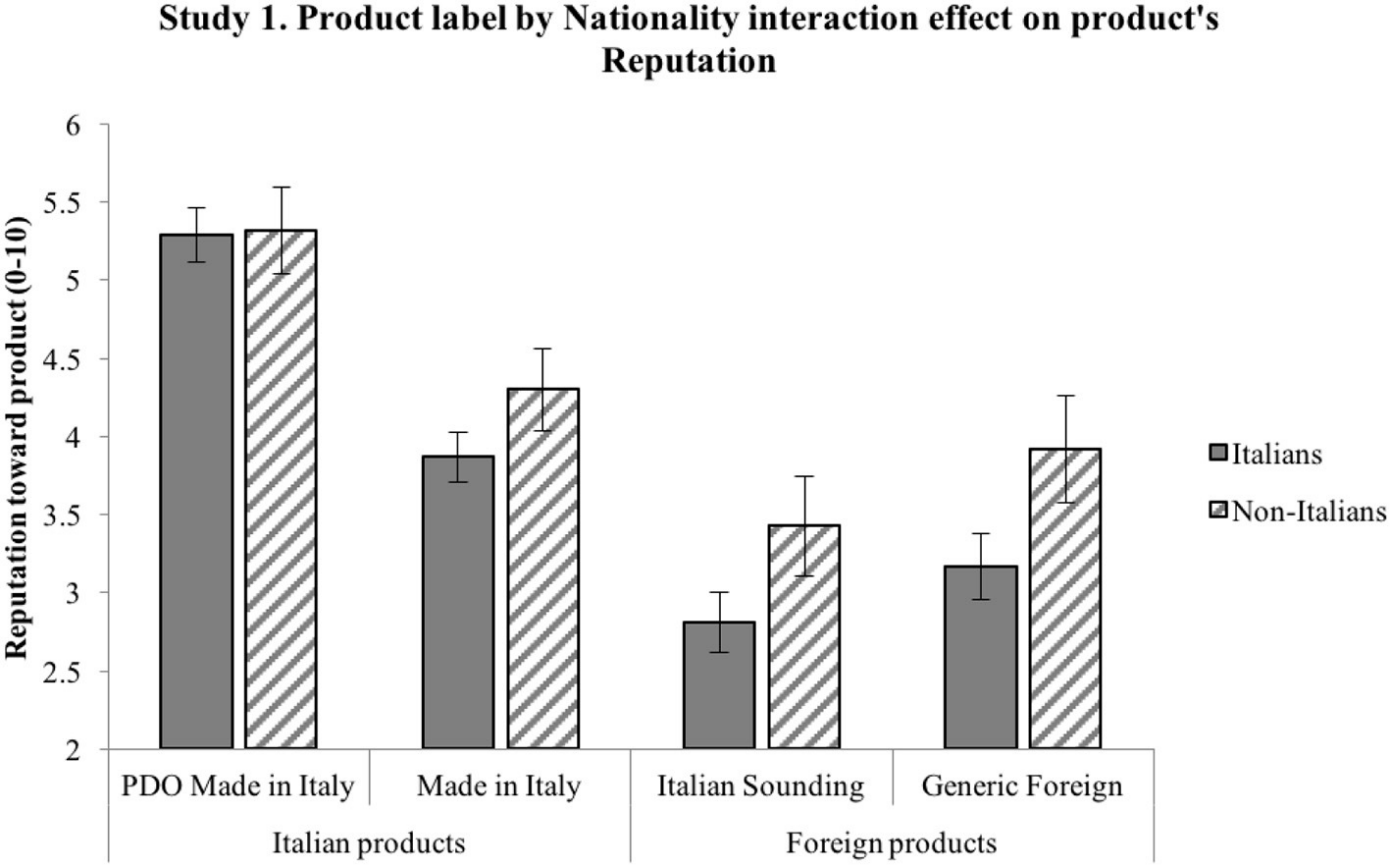
Interaction effect of product label and nationality on product's reputation. Reputation toward product is measured on a scale from 1 (negative reputation) to 10 (positive reputation). Error bars represent 95% confidence interval of the mean. All significant differences have a *p* < 0.001.

**Figure 2:**
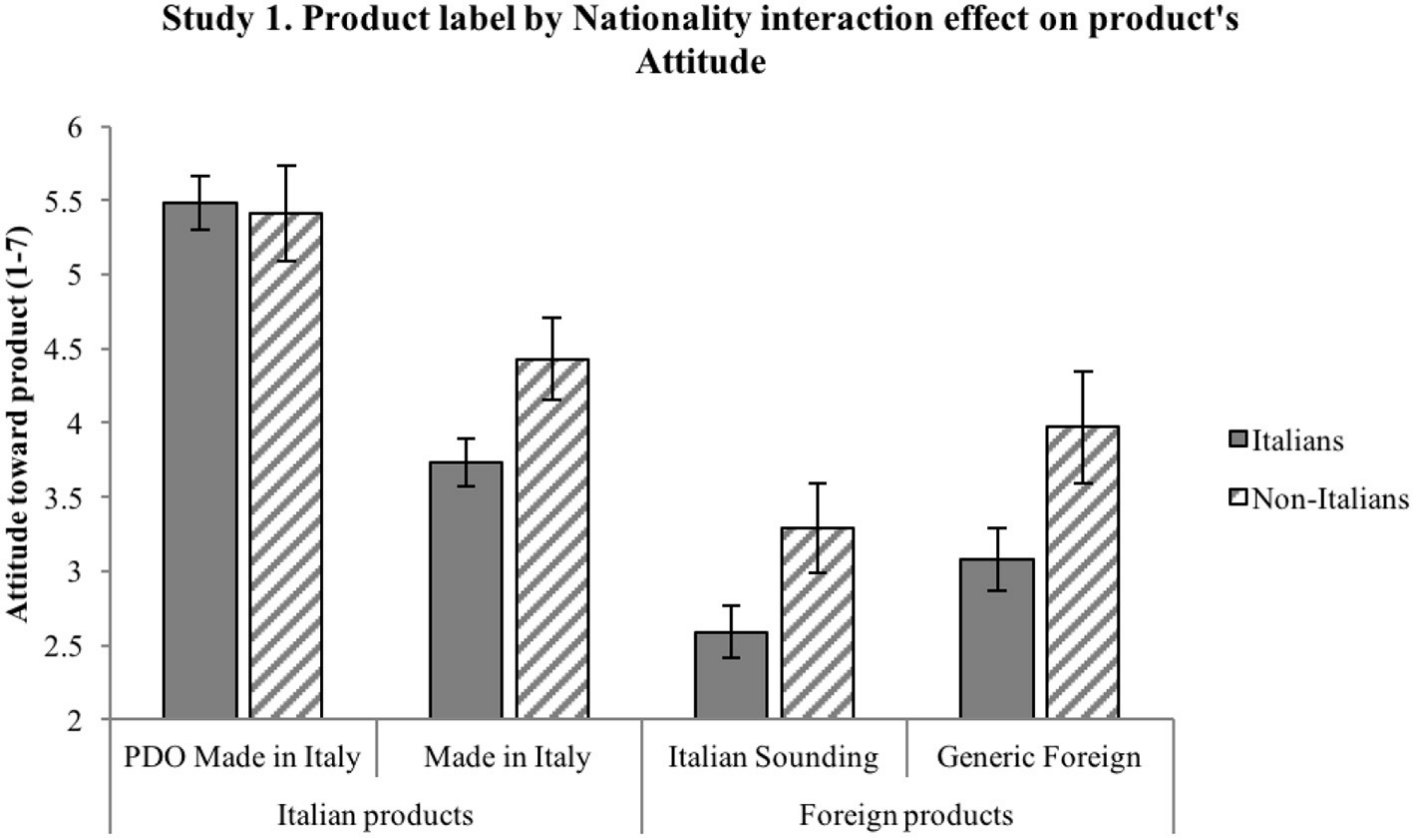
Interaction effect of product label and nationality on attitude toward product. Attitude toward product is measured on a scale from 1 (negative attitude) to 7 (positive attitude). Error bars represent 95% confidence interval of the mean. All significant differences have a *p* < 0.001.

**Figure 3:**
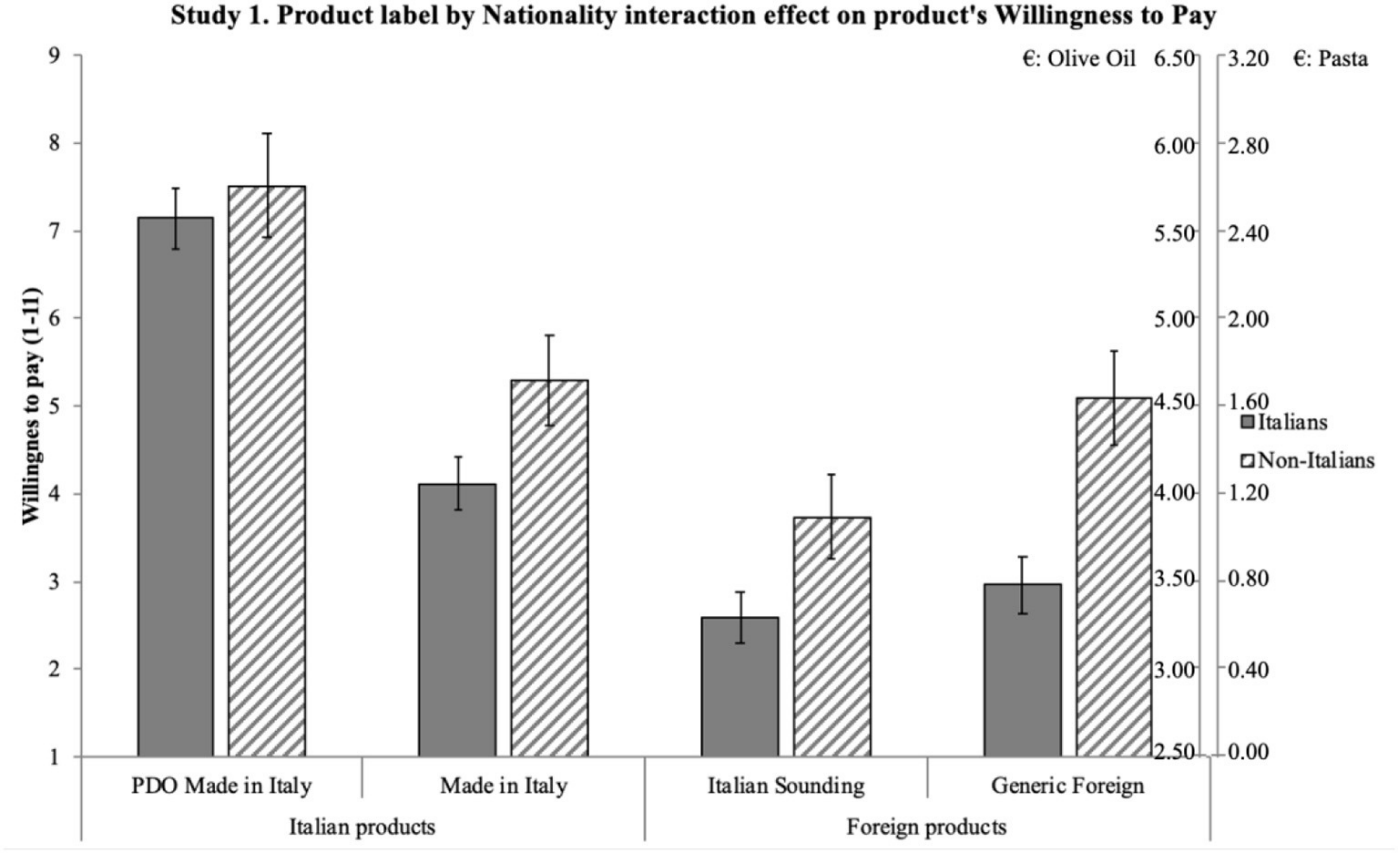
Interaction effect of product label and nationality on WTP. WTP for the product is measured from 1 (WTP a lower price) to 11 (WTP a higher price). WTP ranged from €2.50 to €7.50 for olive oil (with one step increase in the Likert scale corresponding to an increase of €0.50) and from €0.00 to €4.00 for pasta (with one step increase in the Likert scale corresponding to an increase of €0.40). Error bars represent 95% confidence interval of the mean. All significant differences have a *p* < 0.001.

*H1*. ANOVA shows a significant effect of product label on reputation [*F*_(3, 597)_ = 130.57, *p* < 0.001] and a significant effect of nationality on reputation [*F*_(1, 199)_ = 19.97, *p* = 0.001]. Importantly, an interaction effect also emerges between the two independent variables on reputation [*F*_(3, 597)_ = 3.64, *p* = 0.013, η*p*^2^ = 0.018]. Results emerging from the pairwise comparisons are synthetized in Figure 1: significant differences are shown by the 95% intervals of the mean values. Overall, results confirm H1: product label has an effect on reputation such that it is more positive for the Italian products (particularly PDO) compared to both the Italian Sounding and the Generic Foreign products; moreover, PDO Made in Italy's positive reputation is stronger than that of Made in Italy. Non-Italians attribute a more positive reputation than Italians to IS products, as well as to Made in Italy and Foreign products, while the two sub-samples do not differ in their reputational assessment of the PDO Made in Italy products.
*H2*. ANOVA shows a significant effect of product label on attitude [*F*_(3, 540)_ = 146.77, *p* < 0.001] and a significant effect of nationality on attitude [*F*_(1, 180)_ = 27.02, *p* < 0.001]. An interaction effect emerges between the two independent variables on attitude [*F*_(3, 540)_ = 5.75, *p* = 0.001, η*p*^2^ = 0.031]. Results emerging from the pairwise comparisons are synthetized in Figure 2: significant differences are shown by the 95% intervals of the mean values. Overall, results confirm H2: product label has an effect on attitude such that it is more positive for the Italian products (particularly PDO) compared to both the Italian Sounding and the Generic Foreign products; moreover, PDO Made in Italy's positive attitude is stronger than that of Made in Italy. Non-Italians express a more positive attitude than Italians to IS products, as well as to Made in Italy and Foreign products, while the two sub-samples do not differ in their attitudinal assessment of the PDO Made in Italy products.
*H3*. First, a three-way repeated-measures ANOVA (product label × nationality × product type) was run to account for a possible interaction effect due to the price difference in the two selected types of products (olive oil and pasta), which indeed was not significant [*Wilks' Lambda* (3, 194) = 0.98, *p* = 0.40)]. Therefore, the scheduled analysis was conducted. The mixed-model ANOVA shows a significant effect of product label on WTP [*F*_(3, 594)_ = 175.71, *p* < 0.001] and a significant effect of nationality on WTP [*F*_(1, 198)_ = 7.32, *p* < 0.001]. An interaction effect also emerges between the two independent variables on WTP [*F*_(3, 594)_ = 7.13, *p* = 0.001, η*p*^2^ = 0.035]. Results emerging from the pairwise comparisons are synthetized in Figure 3: significant differences are shown by the 95% intervals of the mean values. Overall, results confirm H3: product label has an effect on WTP such that it is higher for the Italian products (particularly PDO) compared to both the Italian Sounding and the Generic Foreign products; moreover, PDO Made in Italy's WTP is higher than that of Made in Italy. Non-Italians declare a greater WTP than Italians for IS products, as well as for Made in Italy and Foreign products, while the two sub-samples do not differ in their WTP for the PDO Made in Italy products.

### Auxiliary Analysis

**Figure 4:**
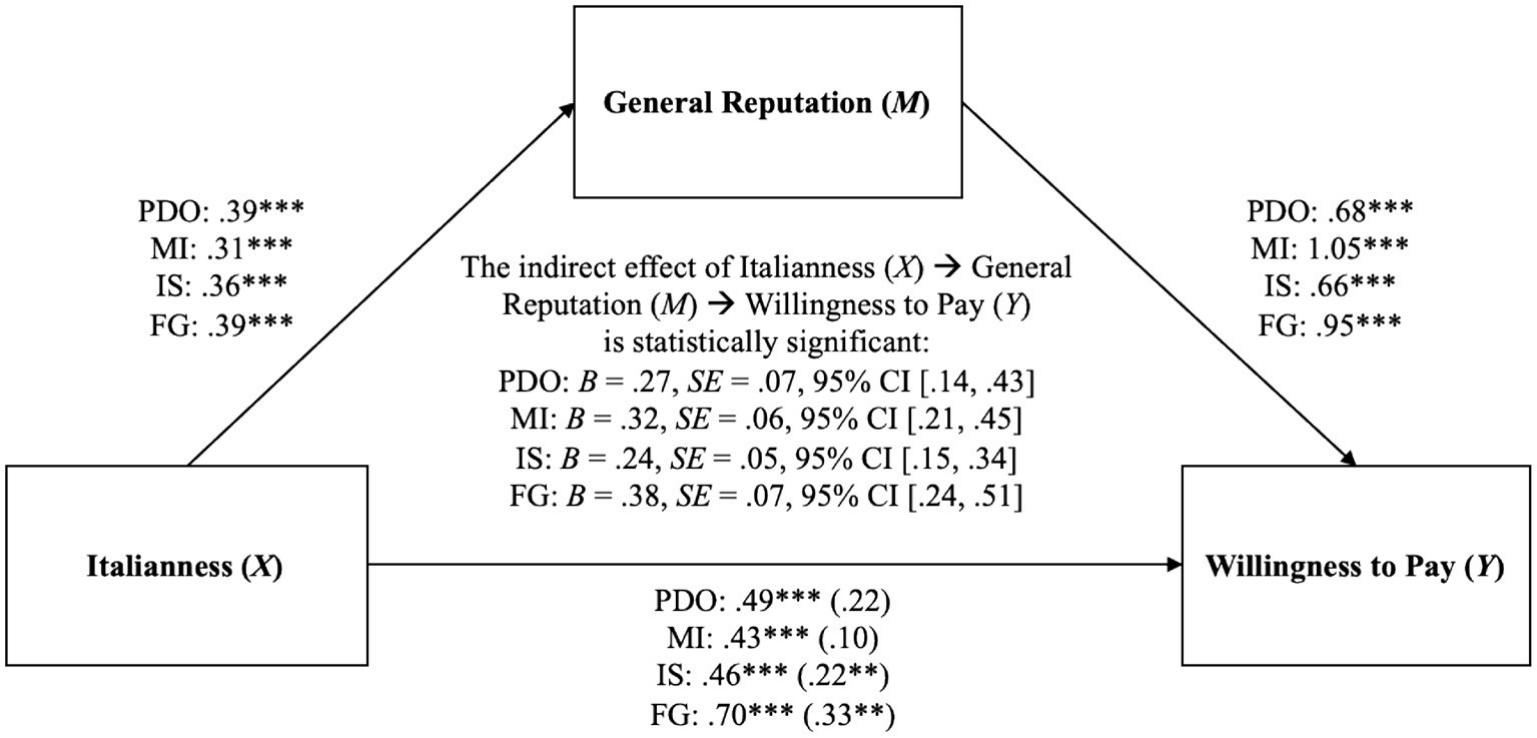
Indirect effects of Italianness (X) on WTP (Y) through General Reputation (M) using bias-correcting bootstrapping (resampled 5,000 times) for each of the four product labels, in Study 1 (Italian and non-Italian in Italy). Values represent standardized estimates. When modeling the relationship between Italianness (X) and WTP (Y), total effects are shown outside parentheses and direct effects are displayed inside parentheses. PDO, PDO Made in Italy; MI, Made in Italy; IS, Italian Sounding; GF, Generic Foreign. ^**^p < 0.01; ^***^p < 0.001.

Given the significant label effect for WTP, a series of four exploratory mediation analyses were conducted. Although differences in Italianness were initially examined as a manipulation check, the follow-up analyses were implemented to test the indirect effect of Italianness on WTP, mediated by reputation. The Italianness score was computed by averaging the Italianness intensity and Italianness probability scores for each product label. The PROCESS Macro for SPSS (Model 4) was used in these analyses (Hayes, 2012). Results (Table 3; Figure 4) support the mediation interpretation: Italianness increased reputation, which was associated with an increase in WTP. The indirect effect of Italianness on WTP via reputation was significant for all the different product labels. Overall, these results suggest that the more any food product (oil or pasta) is perceived to be Italian, the more its reputation will increase, which, in turn, will increase consumers' WTP for that product.

**Table 3:** Summary of mediation analyses predicting Willingness to Pay in the whole sample in Study 1 (Italian and non-Italian in the EU).

**Product label (N)**	**Effect of X on M**	**Effect of M on Y**	**Total effect of X on Y**	**Direct effect of X on Y**	**Indirect effect of X on Y**
	*a*	*b*	*c*	*c'*	*ab* [95% CI]
PDO made in Italy (202)	0.39^***^	0.68^***^	0.49^***^	0.22	0.27^***^ [0.14, 0.43]
Made in Italy (201)	0.31^***^	1.05^***^	0.43^***^	0.1	0.32^***^ [0.21, 0.45]
Italian sounding (200)	0.36^***^	0.66^***^	0.46^***^	0.22^**^	0.24^***^ [0.15, 0.34]
Generic foreign (196)	0.39^***^	0.95^***^	0.70^***^	0.33^**^	0.38^***^ [0.24, 0.51]

*** *p < 0.001*.

*a: effect of X on M; b: effect of M on Y; c: total effect of X on Y; c': direct effect of X on Y; ab: indirect effect of X on Y*.

### Discussion

The three hypotheses were generally confirmed by the results. Reputation, attitude, and WTP differ significantly for the four product labels, with further differences in the three dependent variables when comparing the Italian and non-Italian sub-samples. H1, H2, and H3 have been largely confirmed, as different reputation, attitude, and WTP emerged for the four different product labels: the highest reputation, attitude, and WTP were found for the PDO Made in Italy product, followed by the Made in Italy product, then by the Generic Foreign product, and finally by the Italian Sounding product. To better understand the magnitude of the effect confirming H3, it is worth reverting the Likert values of the WTP scale in euro: critically, for the *DPO Made in Italy* product, subjects have been found willing to pay 10% more than the average price (€5.50 for oil, €2.00 for pasta) of one product item (around €0.50 more for olive oil and €0.40 more for pasta); for the *Made in Italy* product, they would pay 11% less than the average price (around €0.50 less for olive oil and €0.40 less for pasta); for the *IS* product, they would pay 20% less than the average price (around €1.00 less for olive oil and €0.80 less for pasta); for the *Generic Foreign* product, they would pay 16% less than the average price (around €0.75 less for olive oil and €0.60 less for pasta). Overall, results are thus consistent with expectations, as they show a significant effect of perception, in terms of reputation and attitude, on consumers' WTP (Landon and Smith, 1998; Loureiro and McCluskey, 2000; Loureiro and Umberger, 2007).

Also, differences within the two sub-samples were in line with expectations. Confirming H1, H2 and H3, different reputation, attitude, and WTP emerged for three different product labels (Made in Italy, IS, and Generic Foreign), with non-Italian participants reporting significantly higher reputation, attitude, and WTP. Importantly, there was no significant differences in reputation, attitude, and WTP toward the PDO Made in Italy product label between the non-Italian sub-sample and the Italian sub-sample, suggesting that the PDO Made in Italy product is perceived to be the best one by Italians and Europeans alike. Interestingly, Italian Sounding items (oil and pasta) turn out to be the worst ones among the four product labels, contrary to the expectation of their capability to endorse a competitive advantage when compared to the Generic Foreign corresponding ones: such a lack of IS effect can be interpreted within the specific samples and contexts, namely, Italians living in Italy and non-Italians living in the EU (i.e., a close-by target with respect to the country of origin on which the IS phenomenon is based).

Finally, the auxiliary analysis provided critical insights into the psychological process by which a given product, when perceived to be Italian, might gain a financial competitive advantage over other products. Basically, the more a food product is perceived to be Italian, the more positive its reputation is. The higher the reputation, the more its consumers are willing to pay for that given product. The Italianness economic effect of a product is thus critically mediated by its reputational advantage, in both Italian and EU consumers.

Overall, although some caution in the interpretation of results should be used given the different sample sizes of the two considered sub-samples, the main pattern of results suggests that Made in Italy products (especially being PDO Made in Italy, partly being simply Made in Italy) are perceived to be better products than foreign products (being Italian Sounding or Generic Foreign), a crucial insight for Italian products market potential in Italy and abroad. Furthermore, the auxiliary results shed light on the reason why the competitive advantage of Italian products might occur: our results show that it is not Italianness *per se* that directly translates into market value; instead, the reputation gain associated with it is the crucial driver. The next step would be to understand whether this process specifically holds true for Italian Sounding products: in fact, those products that sound Italian but in fact are not (that is, Italian Sounding products) might hold the competitive advantage over other foreign products by “stealing” the reputation of Italian products. This effect, underpinning the Italian Sounding phenomenon, could be even more likely to occur in markets where finding Italian products is not as easy as it is in Italy and in Europe. Indeed, the perception and the WTP for products associated with Italy should be investigated not only in an Italian and EU context, as it was in Study 1. Therefore, research in other continents should be carried out to clarify this issue. Furthermore, the methodology of Study 1 has not included a standard tool to assess more in detail the food reputation profile of the investigated products (over and above a measure of general reputation). This possibility should be included too in the next steps of the research, in order to clarify which peculiar aspects of food reputation are key to explain the Italian Sounding phenomenon. Accordingly, these steps will be addressed by Study 2 first, and then by Study 3. The next two studies will also deepen the interplay among the expected Italian Sounding phenomenon with regard to different contexts and samples: rather than assessing its effects within Italy with Italian and EU samples (as in Study 1), they will move such a test to both China (Study 2) and the USA (Study 3), by thus expecting a much more salient scenario to test the effects hypothesized by the Italian Sounding phenomenon. In Study 2, this issue will be tested more thoroughly by adding a comparison across different non-Italian cultural groups within the same country (i.e., a Chinese vs. a non-Chinese sample).

## Study 2

### Aim and Hypotheses

The main aim of Study 2 is to confirm and enlarge the findings that emerged in Study 1, that is, to investigate how non-Italian subjects perceive, in terms of reputation and attitude, and are willing to pay for an agro-food product associated to Italy (pasta) presented in three forms, differentiated by label (*Made in Italy, Italian Sounding*, and *Generic Foreign*), in a different linguistic and cultural context. In order to shed light on the process by which Made in Italy and Italian Sounding products can gain a competitive advantage on foreign products and to deepen the knowledge of Italian Sounding effects within one of the main global markets, the study has been conducted on a first sample of Chinese citizens (Huliyeti et al., 2008; Vianelli et al., 2012a), as well as on a second sample of non-Italian expatriates in China (to check for the hypothesized effects in the same cultural place but on a different cultural group). In general, the first sample (Chinese citizens in China) is expected to be vulnerable to the Italian Sounding effects (i.e., Italian Sounding product perceived and treated similarly to the Made in Italy one), while the second sample (non-Italian expatriates in China) is expected to be less vulnerable to the Italian Sounding effects (i.e., Italian Sounding product perceived and treated at a lower lever compared to the Made in Italy ones). The PDO product was not included for the first sample, as PDO, being an EU labeling system, is estimated to be meaningful in the EU rather than in Asia (therefore for the second sample only in Study 2); the PDO product is however included in the second sample as expatriates may have a better knowledge of the difference among PDO and non-PDO products that belong from the same country of origin (and also to further test the specific result previously obtained in Study 1, now with a non-Italian sample abroad). Study 2 also deepens the general knowledge acquired with Study 1's findings by measuring more detailed reputation profiles of the products via a list of items reproducing the main 23 features emerged in the *Food Reputation Map* (FRM, Bonaiuto et al., 2017; De Dominicis et al., 2020).

It is thus expected that:

*H4:* The product form or label has an effect on reputation profiles measured via FRM, which are more positive for *Made in Italy* and *Italian Sounding* products compared to the *Generic Foreign Chinese* product in the first sample (Chinese in China), while in the second sample (Expatriates in China), the *PDO Made in Italy* product is the highest, the *Made in Italy* product is the second highest, and the other two products are the lowest.
*H5:* The product has an effect on general reputation: In particular, reputation is more positive for *Made in Italy* and *Italian Sounding* products compared to the *Generic Foreign Chinese* product in the first sample (Chinese in China), while in the second sample (Expatriates in China), the *PDO Made in Italy* product is the highest, the *Made in Italy* product is the second highest, and the other two products are the lowest ones.
*H6:* The product has an effect on the attitude: In particular, the attitude is more positive for *Made in Italy* and *Italian Sounding* products compared to the *Generic Foreign* product in the first sample (Chinese in China), while in the second sample (Expatriates in China), the *PDO Made in Italy* product is the highest, the *Made in Italy* product is the second highest, and the other two products are the lowest ones.
*H7:* The product has an effect on the WTP: In particular, WTP is higher for *Made in Italy* and *Italian Sounding* products compared to the *Generic Foreign Chinese* product in the first sample (Chinese in China), while in the second sample (Expatriates in China), the *PDO Made in Italy* product is the highest, the *Made in Italy* product is the second highest, and the other two products are the lowest ones.

### Method

#### Participants, Procedure, and Materials

Data were collected on two samples. The first sample is composed of 100 subjects of Chinese nationality: 56% were women, 48% were 18–25 years old, 35% were 26–34 years old, 13% were 35–44 years old, and 4% were 45–54 years old. The second sample is composed of 91 non-Italian expatriates in China (living in China and not an Italian or Chinese nationality): 42% were women; 49.5% were 18–25 years old, 39.6% were 26–34 years old, 8.8% were 35–44 years old, 2.2% were 45–54 years old; nationality: 22% USA, 15.4% Germany, 12.1% Malaysia, 8.8% Singapore, 6.6% North Korea, 4.4% Iran, 4.4% Switzerland, and 26.3% other countries. The questionnaire for the two samples was administered electronically in May 2015 via the major Chinese social network (WeChat) and via networks such as “CrackingChina” and “ExpatMix.” In order to identify which products could be investigated in a context that is culturally different from the Italian one, a similar pre-test as in Study 1 was conducted on 20 subjects selected at Zhejiang University in Hangzhou (asking a preliminary sample to indicate two information: the most known and the most consumed Italian food product). The pre-test results and calculated count of the subjects' responses show that the best known and most consumed Italian product in China is pasta. For the first sample, the questionnaire was translated from English to Mandarin Chinese with the collaboration of a group of master's and PhD students from Zhejiang University, and a back-translation was carried out. As in Study 1, for the first sample, each subject was asked to fill in a questionnaire, observing the images of the three different products reported in it, presented in the following order: *Made in Italy, Italian Sounding*, and *Generic Foreign Chinese*; for the second sample, each subject was asked to fill in a questionnaire, observing the images of the four different products reported in it, presented in the following order: *PDO Made in Italy, Made in Italy, Italian Sounding*, and *Generic Foreign Chinese*.

The three selected products were as follows (again, as in Study 1, avoiding major brands): “Spaghetti Capellini Agnesi” as *Made in Italy*, “Spaghetti San Remo” as *Italian Sounding*, and one Chinese spaghetti, as *Generic Foreign Chinese* (Appendix B); for the second sample only, the fourth product was a *PDO Made in Italy* (“Spaghetti Gentile Pasta di Gragnano”).

#### Measures

The questionnaire is similar to the one used in Study 1, although only one version was produced, as only one type of product (pasta) was explored, indicated as the most representative of the Italian cuisine by the pre-test. The questionnaire, investigating three product forms (*Made in Italy, Italian Sounding*, and *Generic Foreign Chinese*), was administered in Mandarin Chinese to Chinese citizens in China for the first sample; moreover, the questionnaire, investigating four product forms (*PDO Made in Italy, Made in Italy, Italian Sounding*, and *Generic Foreign Chinese*) was administered in English to non-Italian expatriates in China for the second sample. The whole survey is available in the Supplementary Material of this manuscript.

The same three types of manipulation checks as Study 1 were used to measure Italianness intensity, probability, and origin.

To measure the product's general reputation, the same one-item seven-point Likert-type scale as Study 1 was used. Reputation profiles of each product were investigated via a new 23-item set on a seven-point Likert-type scale (from “strongly disagree” to “strongly agree”), created *ad hoc* by adapting the 23 indicators of the FRM (Bonaiuto et al., 2017; De Dominicis et al., 2020). Four items measured *Essence*; four items measured *Cultural Effects*; three items measured *Economic Effects*; four items measured *Environmental Effects*; three items measured *Physiological Effects*; five items measured *Psychological Effects* (see Appendix C).

The same 10 seven-point evaluative semantic differential scales as Study 1 were used to investigate attitude by means of the same bi-polar couples of adjectives.

To measure WTP, the same one-item 11-point Likert-type scale (adapted from Hanemann, 1984) as Study 1 was used, expressing prices in yuan, ranging from “¥0” to “¥40” for the first sample (¥4 cumulative increase in each step) and in US dollars (from $0 to $3) for the second sample ($0.30 cumulative increase in each step), where the scale's middle point was close to a possible national average price (¥20 or $1.50) for the product in that period.

For the non-Italian expatriates in China sample, the “pasta” item was: “Considering that the average price of a 500 g of pasta is about $1.50, how much would you be willing to pay if you would buy 500 g of PRODUCT NAME”; for the Chinese sample, the item was the same one written in Chinese Mandarin language.

As for Study 1, all statistical analyses were released using the SPSS version 27 software.

#### Manipulation Check

Similarly to Study 1, a series of manipulation checks was performed to test whether the manipulation of the products' form or label, being PDO Made in Italy, Made in Italy, Italian Sounding, or Generic Foreign products, was effective in changing the perception of Italianness (intensity, probability, and origin) in both samples of participants.

On the first sample (Chinese respondents in China), two repeated-measures ANOVAs were performed to test the product label effects on the dependent variable “Italianness intensity” (score 0–10) and “Italianness probability” (0–100%). The manipulation checks indicated an effect of the product label both on Italianness intensity [*F*_(2, 198)_ = 127.86, *p* < 0.001, η*p*^2^ = 0.56] and on Italianness probability [*F*_(2, 198)_ = 86.16, *p* < 0.001, η*p*^2^ = 0.46], such that, as expected, Made in Italy and Italian Sounding products, while not differing from each other, were significantly higher in both dependent variables than the Generic Foreign products (Table 4). Furthermore, three binary logistic regressions were conducted to understand whether Italian origin (yes/no) was predicted by each product label. The Made in Italy and the Italian Sounding products were perceived Italian by the slight majority (59 and 54% of cases, respectively), while Generic Foreign product was considered foreign in 96% of cases (Table 4). Therefore, it is possible to conclude that the manipulation was effective on the Chinese sample, according to the sample-specific expectations: the Made in Italy product and the Italian Sounding product were both perceived as more Italian than the Generic Foreign product.

**Table 4:** Mean scores and *SD* of product label related to Italianness intensity, probability, and origin, for the first sample of Chinese respondents in China (Study 2).

**Product label**	**Italianness intensity**	**Italianness probability**	**Italianness origin**
	**M (SD)**	**M (SD)**	**Italian%/foreign**
	**(N = 100)**	**(N = 100)**	**% (N), sig**.
Made in Italy	6.28 (2.13)	3.32 (1.31)	59/41 (100), *p* = 0.073
Italian sounding	6.68 (2.25)	3.11 (1.27)	54/45 (99), *ns*
Generic foreign	2.36 (2.36)	1.48 (0.91)	4/96 (100), *p* < 0.001

On the second sample (non-Italian expats respondents in China), two repeated-measures ANOVAs were performed to test the effects of product label on the dependent variable “Italianness intensity” (score 0–10) and “Italianness probability” (0–100%). The manipulation checks indicated an effect of the product label both on Italianness intensity [*F*_(2.43, 218.91)_ = 51.76, *p* < 0.001, η*p*^2^ = 0.36] and on Italianness probability [*F*_(3, 270)_ = 37.42, *p* < 0.001, η*p*^2^ = 0.29], such that, as expected, both dependent variables significantly decreased from PDO Made in Italy to Made in Italy to Italian Sounding to Generic Foreign products. Estimated marginal means comparisons showed significant differences across all four means (see values in Table 5) in each of the two dependent variables. Furthermore, three binary logistic regressions were conducted to understand whether Italian origin (yes/no) was predicted by each product label. All four models were statistically significant (all *p* < 0.001) and predicted Italian (or non-Italian) origin as expected: the PDO Made in Italy and Made in Italy products were considered Italian products in 97 and 74% of cases, respectively, while the Italian Sounding and Generic Foreign products were considered foreign products in 89.7 and 95.3% of cases, respectively. Therefore, it is possible to conclude that the manipulation was effective, again according to the sample-specific expectations: the PDO Made in Italy product was perceived as more Italian than the Made in Italy product, which, in turn, was perceived as more Italian than the Italian Sounding product, which finally was perceived more Italian than the Generic Foreign product.

**Table 5:** Mean scores and *SD* of product label related to Italianness intensity, probability, and origin, for the second sample of non-Italian expats respondents in China (Study 2).

**Product label**	**Italianness intensity**	**Italianness probability**	**Italianness origin**
	**M (SD)**	**M (SD)**	**Italian%/foreign%**
	**(*N* = 91)**	**(*N* = 91)**	**(*N*), sig**.
PDO made in Italy	7.82 (1.66)	3.93 (0.96)	83.5/16.5 (91), *p* < 0.001
Made in Italy	6.70 (1.79)	3.52 (1.24)	76.9/23.1 (91), *p* < 0.001
Italian sounding	5.18 (2.44)	3.12 (1.21)	59.3/40.7 (91), *p* = 0.076
Generic foreign	4.60 (2.66)	2.24 (1.20)	20.9/79.1 (91), *p* < 0.001

### Results

#### Comparison of Indicators of Food Reputation Across Products (H4)

Results are separately reported for the first and the second sample. Regarding the first sample, to test H4, a series of repeated-measures ANOVA was conducted on each of the 23 specific indicators of the FRM, comparing each indicator across the products. The results of the repeated-measures ANOVAs, *post hoc* comparisons, and descriptive statistics are presented in Table 6 by grouping them into the six areas identified by the synthetic indicators of FRM: *Essence, Cultural Effects, Economic Effects, Environmental Effects, Physiological Effects*, and *Psychological Effects*.

**Table 6:** Mean (*M*) and Standard Deviation (*SD*) scores for 23 reputation features related to the three product labels and the relevant *p*-value indicating the statistical significance of each difference (ANOVA), for the first sample (Chinese citizens in China).

	**Omnibus effect**	**M (SD)**			**Significance (*****p*****-value)**		
	***F(df)*** **ηp^**2**^**	**Made in Italy** **(MI)**	**Italian sounding** **(IS)**	**Generic Foreign Chinese** **(GFC)**	**MI-IS**	**MI-GFC**	**IS-GFC**
**ESSENCE**							
Composition	0.83 (2, 198) 0.43	4.43 (1.11)	4.31 (1.14)	4.26 (1.04)	1.000	0.569	1.000
Genuineness	0.32 (1.63, 161.19) 0.00	4.16 (1.27)	4.25 (1.27)	4.11 (1.70)	1.000	1.000	1.000
Life time	1.97 (2, 198) 0.02	5.05 (1.17)	4.81 (1.28)	5.10 (1.24)	0.250	1.000	0.248
Recognition	3.42 (2, 198)^*^ **0.03**	4.73 (1.12)	4.53 (1.24)	4.30 (1.53)	0.524	**0.054**^**∧**^	0.514
**CULTURAL EFFECTS**							
Territorial identity	4.31 (2, 198)^*^ **0.04**	4.87 (1.20)	4.61 (1.37)	4.33 (1.62)	0.390	0.012^*^	0.478
Tradition	5.61 (2, 198)^**^ **0.05**	4.80 (1.18)	4.61 (1.21)	4.28 (1.51)	0.590	0.006^**^	0.129
Familiarity	1.10 (2, 198) 0.01	4.67 (1.36)	4.54 (1.21)	4.40 (1.51)	1.000	0.420	1.000
Innovativeness	27.37 (1.82, 179.87)^***^ **0.22**	4.45 (1.27)	4.22 (1.23)	3.23 (1.61)	0.352	0.000^***^	0.000^***^
**ECONOMIC EFFECTS**							
Context	14.97 (2, 198)^***^ **0.13**	4.49 (1.32)	4.53 (1.16)	3.67 (1.42)	1.000	0.000^***^	0.000^***^
Price	3.04 (2, 198)^*^0.03	4.38 (1.18)	4.46 (1.23)	4.75 (1.36)	1.000	**0.080**^**∧**^	0.260
Preparation	6.57 (2, 198)^**^ **0.06**	4.94 (1.07)	4.83 (0.93)	5.31 (1.38)	1.000	0.032^*^	0.005^**^
**ENVIRONMENTAL EFFECTS**							
Social and environmental responsibility	0.94 (2, 198) 0.01	4.65 (1.21)	4.83 (1.16)	4.63 (1.33)	0.770	1.000	0.660
Traceability	7.39 (1.79, 177.3)^**^ **0.07**	4.61 (1.24)	4.50 (1.01)	4.02 (1.54)	1.000	0.004^**^	0.022^*^
Proximity	0.98 (2, 198)0.01	4.41 (1.40)	4.62 (1.12)	4.42 (1.53)	0.626	1.000	0.681
Safety	**2.98 (2, 198)**^**∧**^ **0.03**	4.82 (1.23)	4.59 (1.20)	4.43 (1.39)	0.410	**0.051**^**∧**^	1.000
**PHYSIOLOGICAL EFFECTS**							
Ability to Satisfy	9.80 (2, 198)^***^ **0.09**	5.27 (1.41)	4.95 (1.34)	5.64 (1.48)	0.115	**0.062**^**∧**^	0.000^***^
Digestibility	0.99 (2, 198) 0.01	4.75 (1.39)	4.90 (1.37)	4.99 (1.56)	0.997	0.526	1.000
Lightness	**6.72 (1.86, 184.33)** ^**^ **0.06**	3.95 (1.34)	4.37 (1.23)	3.80 (1.47)	0.009^**^	1.000	0.003^**^
**PSYCHOLOGICAL EFFECTS**							
Organoleptic perception	9.79 (2, 198)^***^ **0.09**	4.40 (1.38)	4.30 (1.21)	3.74 (1.43)	1.000	0.000^***^	0.004^**^
Personal memories	0.76 (2, 198) 0.01	3.83 (1.54)	4.03 (1.42)	4.05 (1.76)	0.882	0.880	1.000
Psycho-physical well-being	1.38 (2, 198) 0.01	4.06 (1.46)	4.07 (1.24)	3.83 (1.44)	1.000	0.589	0.471
Conviviality	21.61 (2, 198)^***^ **0.18**	4.17 (1.42)	4.18 (1.45)	3.22 (1.53)	1.000	0.000^***^	0.000^***^
Group belongingness	2.13 (2, 198) 0.02	4.00 (1.50)	4.00 (1.53)	3.67 (1.66)	1.000	0.250	0.260

*Bold values represents significant or marginally significant effects*.

∧ *p < 0.10*;

* *p < 0.05*;

** *p < 0.01*;

*** *p < 0.001*.

As for the *Essence* scores, the products do not significantly differ (except for *Made in Italy* enjoying a tendency to a higher *Recognition* than *Generic Foreign Chinese*).

As for *Cultural Effects*, all dimensions contribute to defining the products' distinctive reputation profile, except for *Familiarity*. In particular, both *Territorial identity* and *Tradition* mark a positive difference of the *Made in Italy* product compared to the *Generic Foreign Chinese* product. *Innovativeness* is common to *Made in Italy* and *Italian Sounding* products and positively differentiates them from the *Generic Foreign Chinese* product.

As for *Economic Effects*, both *Context* and *Preparation* differentiate the profile of the *Generic Foreign Chinese* product from both the *Made in Italy* and the *Italian Sounding* ones, which do not differ from each other: while on the first variable they are more positive, on the second variable, they are less positive than the *Generic Foreign Chinese*. Moreover, the *Generic Foreign Chinese* product enjoys a more positive *Price* ratio than the Made in Italy product.

As for *Environmental Effects*, results show that *Traceability* positively differentiates both *Made in Italy* and *Italian Sounding* products from the *Generic Foreign Chinese* product; while *Safety* positively differentiates only the Made in Italy one from the *Generic Foreign Chinese* one, *Proximity* does not show significant differences.

As for *Physiological Effects*, the averages obtained from the different products in *Digestibility* are not significantly different, while significant differences emerge with respect to *Ability to Satisfy*—a dimension in which the reputation score of the *Generic Foreign Chinese* product is higher than both the *Italian Sounding* and the *Made in Italy* products, as well as with respect to *Lightness*—for which the reputation of the *Italian Sounding* product is significantly higher than both the *Made in Italy* and the *Generic Foreign Chinese* ones.

As for *Psychological Effects*, results show both *Organoleptic perception* and *Conviviality* positively differentiating the reputation profiles of both *Made in Italy* and *Italian Sounding* products from that of the *Generic Foreign Chinese* product, while neither *Personal memories*, nor *Psycho-physical well-being*, nor *Group belongingness* differentiates among the three products' reputation.

Regarding the second sample, to test H4, a series of repeated-measures ANOVA was then conducted on each of the 23 specific indicators of the FRM, comparing each indicator with the four product forms. The results of the repeated-measures ANOVAs, *post hoc* comparisons, and descriptive statistics are presented in Table 7 by grouping them into the six areas identified by the synthetic indicators of FRM: *Essence, Cultural Effects, Economic Effects, Environmental Effects, Physiological Effects*, and *Psychological Effects*.

**Table 7:** Mean (*M*) and Standard Deviation (*SD*) scores for 23 reputation features related to the three product labels and the relevant *p*-value indicating the statistical significance of each difference (ANOVA), for the second sample (non-Italian expatriates in China).

	**Omnibus effect**	**M (SD)**				**Significance (*****p*** **value)**					
	***F(df)*** **ηp^**2**^**	**PDO** **Made in Italy** **(PMI)**	**Made in Italy** **(MI)**	**Italian Sounding** **(IS)**	**Generic** **Foreign** **Chinese** **(GFC)**	**PMI-MI**	**PMI-IS**	**PMI-GFC**	**MI-IS**	**MI-GFC**	**IS-GFC**
**ESSENCE**											
Composition	19.22 (3, 270)^***^ **0.18**	4.82 (1.23)	4.27 (1.18)	3.69 (1.50)	3.78 (1.29)	0.009^**^	0.000^***^	0.000^***^	0.008^**^	0.041^*^	1.000
Genuineness	19.21 (3, 270)^***^ **0.18**	4.97 (1.23)	4.11 (1.27)	3.74 (1.53)	3.77 (1.46)	0.000^***^	0.000^***^	0.000^***^	0.304	0.460	1.000
Life time	11.64 (2.33, 209.64)^***^ **0.11**	5.19 (1.21)	5.04 (1.29)	4.19 (1.72)	4.44 (1.44)	1.000	0.000^***^	0.000^***^	0.002^**^	0.005^**^	1.000
Recognition	13.76 (3, 270)^***^ **0.13**	4.88 (1.25)	4.68 (1.27)	4.03 (1.49)	4.11 (1.40)	0.951	0.000^***^	0.000^***^	0.001^**^	0.006^**^	0.951
**CULTURAL EFFECTS**											
Territorial identity	26.30 (3, 270)^***^ **0.23**	5.11 (1.18)	4.56 (1.31)	3.59 (1.57)	3.84 (1.33)	0.018^*^	0.000^***^	0.000^***^	0.000^***^	0.001^**^	1.000
Tradition	10.34 (3, 270)^***^ **0.10**	4.63 (1.39)	4.30 (1.35)	3.77 (1.46)	3.69 (1.46)	0.575	0.001^**^	0.000^***^	**0.055**^**∧**^	0.011^*^	1.000
Familiarity	14.97 (3, 270)^***^ **0.14**	4.82 (1.34)	4.59 (1.41)	3.68 (1.40)	4.11 (1.57)	1.000	0.000^***^	0.001^**^	0.000^***^	**0.088**^**∧**^	0.145
Innovativeness	6.94 (2.68, 240.91)^***^ **0.07**	4.35 (1.66)	4.09 (1.66)	3.57 (1.50)	4.31 (1.10)	0.924	0.005^**^	1.000	0.114	1.000	0.000^***^
**ECONOMIC EFFECTS**											
Context	26.26 (3, 270)^***^ **0.23**	4.99 (1.16)	4.20 (1.42)	3.47 (1.41)	3.78 (1.55)	0.000^***^	0.000^***^	0.000^***^	0.000^***^	0.312	0.595
Price	22.33 (2.38, 214.62)^***^ **0.20**	5.15 (1.14)	4.41 (1.27)	4.05 (1.68)	3.68 (1.31)	0.000^***^	0.000^***^	0.000^***^	0.756	0.001^**^	0.308
Preparation	**2.59 (2.48, 223.57)**^**∧**^**0.03**	5.11 (1.49)	5.08 (1.27)	5.32 (1.39)	5.53 (0.89)	1.000	1.000	**0.080**^**∧**^	1.000	0.026^*^	1.000
**ENVIRONMENTAL EFFECTS**											
Social and environmental responsibility	11.01 (3, 270)^***^ **0.11**	4.92 (1.19)	4.49 (1.17)	4.07 (1.36)	4.14 (1.22)	**0.069**^**∧**^	0.000^***^	0.000^***^	**0.078**^**∧**^	0.239	1.000
Traceability	11.36 (3, 270)^***^ **0.11**	5.07 (1.32)	4.58 (1.26)	4.14 (1.30)	4.15 (1.44)	0.024^*^	0.000^***^	0.000^***^	0.150	0.137	1.000
Proximity	4.15 (2.63, 236.92)^**^ **0.04**	4.57 (1.43)	3.95 (1.46)	4.00 (1.44)	4.18 (1.24)	0.008^**^	**0.050**^**∧**^	0.214	1.000	1.000	1.000
Safety	13.87 (2.75, 247.12)^***^ **0.13**	5.14 (1.26)	4.53 (1.24)	4.12 (1.35)	4.21 (1.35)	0.002^**^	0.000^***^	0.000^***^	0.149	0.582	1.000
**PHYSIOLOGICAL EFFECTS**											
Ability to satisfy	5.22 (2.70, 243.31)^**^ **0.05**	4.84 (1.40)	5.03 (1.33)	4.44 (1.40)	4.45 (1.16)	1.000	0.290	**0.066**^**∧**^	0.021^*^	0.024^*^	1.000
Digestibility	7.28 (2.74, 246.84)^***^ **0.07**	4.68 (1.37)	4.44 (1.42)	4.08 (1.38)	4.07 (1.28)	0.527	0.002^**^	0.000^***^	0.197	0.227	1.000
Lightness	6.67 (3, 270)^***^ **0.07**	4.37 (1.45)	3.69 (1.52)	3.68 (1.48)	3.93 (1.40)	0.000^***^	0.002^**^	0.046^*^	1.000	1.000	0.953
**PSYCHOLOGICAL EFFECTS**											
Organoleptic perception	**5.96 (3, 270)** ^**^ **0.06**	4.97 (1.39)	4.36 (1.39)	4.26 (1.36)	4.36 (1.38)	0.003^**^	0.004^**^	0.009^**^	1.000	1.000	1.000
Personal memories	6.33 (2.73, 245.74)^**^ **0.07**	4.40 (1.42)	3.85 (1.53)	3.67 (1.54)	3.71 (1.47)	0.028^*^	0.001^***^	0.000^***^	1.000	1.000	1.000
Psycho-physical well-being	6.64 (2.79, 251.03)^***^ **0.07**	4.27 (1.40)	3.62 (1.43)	3.62 (1.59)	3.70 (1.57)	0.000^****^	0.005^*^	0.002^**^	1.000	1.000	1.000
Conviviality	7.49 (2.44, 219.32)^***^ **0.08**	4.87 (1.18)	4.35 (1.51)	4.04 (1.50)	4.29 (1.16)	0.014^*^	0.001^**^	0.000^***^	0.951	1.000	1.000
Group belongingness	18.10 (2.33, 209.61)^***^ **0.17**	4.90 (1.17)	3.99 (1.62)	3.67 (1.61)	3.88 (1.33)	0.000^***^	0.000^***^	0.000^***^	0.982	1.000	0.982

*Bold values represents significant or marginally significant effects*.

∧ *p < 0.10*;

* *p < 0.05*;

** *p < 0.01*;

*** *p < 0.001*.

As for the *Essence* scores, there are several statistically significant differences among the products. *Italian Sounding* and *General Foreign Chinese* do not differ in these features, while they are both always less reputable when compared to the *PDO Made in Italy*. *Composition* appears to be the one best discriminating among those products, while regarding *Genuineness*, only *PDO Made in Italy* has a significantly higher score compared to all other products. Regarding *Recognition* and *Life time*, the two *Made in Italy* products both enjoy higher scores than the couple represented by the *Italian Sounding* and *General Foreign Chinese* products. Thus, the general pattern is that *PDO Made in Italy*, and often *Made in Italy* too, is better than both *Italian Sounding* and *Generic Foreign Chinese*.

As for *Cultural Effects*, all dimensions contribute to defining the products' distinctive reputation profile. It can be highlighted how the most discriminating feature is the one about *Territorial identity*: the *PDO Made in Italy* product has the best reputation compared to the other three; moreover, the *Made in Italy* product has a better reputation than the *Italian Sounding* and the *Generic Foreign Chinese* one. The *Italian Sounding* product and the *Generic Foreign Chinese* one never differ in any dimension within this area (*Tradition* and *Familiarity*), except the *Innovativeness* feature, where the *Italian Sounding* product reports the lowest score compared to both *PDO Made in Italy* and the *Generic Foreign Chinese* one. Moreover, the *PDO Made in Italy* and the Made in Italy products do not differ in *Tradition, Familiarity*, and *Innovativeness*. Thus, the general pattern for *Cultural Effects* is that both *PDO Made in Italy* and *Made in Italy* are very often better than both *Italian Sounding* and *Generic Foreign Chinese*.

As for *Economic Effects*, the *PDO Made in Italy* product receives higher scores compared to all other products for *Context* and for *Price*; the *Made in Italy* product partly enjoys a more positive reputation than the *Italian Sounding* (for *Context*) and the *Generic Foreign Chinese* ones (*Price*). Moreover, similarly to the first sample, the feature *Preparation* is more positive in the *Generic Foreign Chinese*, in this case compared to both *Made in Italy* and (tendency) *PDO Made in Italy*. Thus, the pattern for *Economic Effects* is articulated: *PDO Made in Italy* is better than both *Italian Sounding* and *Generic Foreign Chinese* only for Context, while *Italian Sounding* equals *Made in Italy* in Price and *Generic Foreign Chinese* equals *Made in Italy* in Context, and it overrides both *Made in Italy* products in *Preparation*.

As for *Environmental Effects*, the *PDO Made in Italy* product reports significantly higher scores compared to all three other products both for *Traceability* and for *Safety*, as well as for *Social and environmental responsibility* (though with only a tendency for *Made in Italy*); moreover, *PDO Made in Italy* enjoys a more positive reputation for *Proximity* as compared to both *Made in Italy* and *Italian Sounding*. The *Made in Italy* product here enjoys a more positive reputation only compared to the *Italian Sounding* and only on *Social and environmental responsibility*. Thus, the general pattern for *Environmental Effects* is that *PDO Made in Italy* is very often better than both *Italian Sounding* and *Generic Foreign Chinese*.

As for *Physiological Effects*, it is on the *Lightness* that the *PDO Made in Italy* has the best reputation compared to all other three products. Moreover, for *Digestibility*, the *PDO Made in Italy* has a more positive reputation compared to both *Italian Sounding* and *Generic Foreign Chinese* ones, but not compared to the *Made in Italy* one, which, however, has a more positive reputation than those two in terms of *Ability to satisfy*. Thus, the general pattern for *Physiological Effects* is that either *PDO Made in Italy* or *Made in Italy*—although with different peculiarities (*Lightness* and *Digestibility* for *PDO Made in Italy, Ability to satisfy* for *Made in Italy*)—is better than both *Italian Sounding* and *Generic Foreign Chinese*.

As for *Psychological Effects, PDO Made in Italy* consistently reports the highest reputation score, compared to all three other products, in each feature, namely, *Organoleptic perception, Personal memories, Psycho-physical well-being, Conviviality*, and *Group belongingness*.

Thus, the general pattern for *Psychological Effects* is that the *PDO Made in Italy* product enjoys the best reputation in all features (*Organoleptic perception, Personal memories, Psycho-physical well-being, Conviviality*, and *Group belongingness*) as compared to any other product, namely, both *Made in Italy* and *Italian Sounding* and *Generic Foreign Chinese*.

#### Comparison of General Reputation, Attitude, and WTP Across Products (H5–H6–H7)

**Figure 5:**
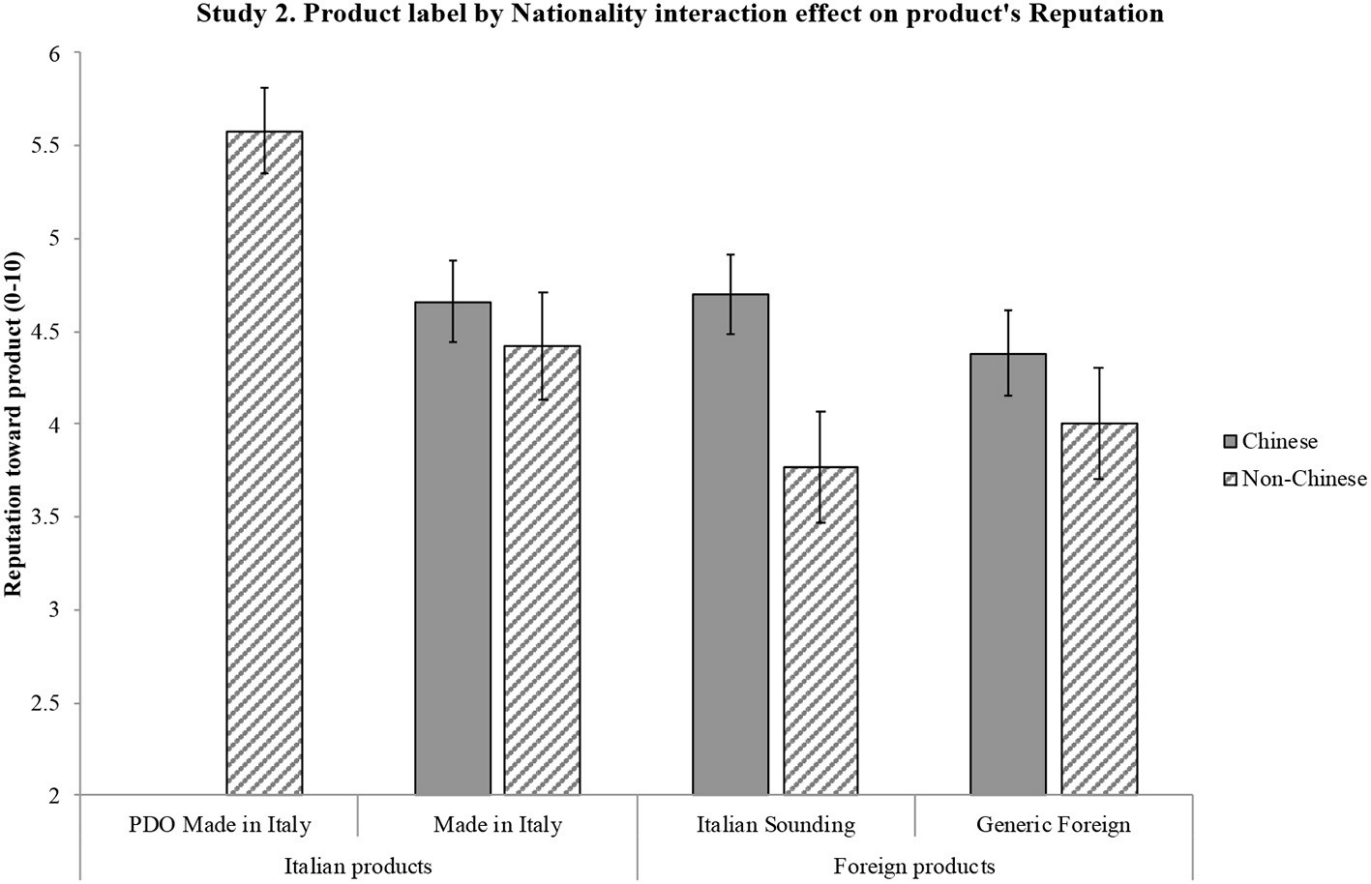
Interaction effect of product label and nationality on product's reputation. Reputation toward product is measured on a scale from 1 (negative reputation) to 10 (positive reputation). Error bars represent 95% confidence interval of the mean. All significant differences have a *p* < 0.001. For visualization purposes, all means from the two sub-samples are plotted together; yet, pairwise comparisons are made across products within the same sample.

Results are separately reported for the first (Chinese) and the second (non-Chinese expatriates) sample. Regarding the first sample (Chinese), to test H5, a repeated-measures ANOVA of products on the general reputation scores was conducted, showing a significant omnibus effect, *F*_(2, 198)_ = 2.98, *p* = 0.05, η*p*^2^ = 0.03. The subsequent *post hoc* comparisons show that the reputation mean scores of *Made in Italy* (*M* = 4.66, *SD* = 1.10) and *Italian Sounding* (*M* = 4.7, *SD* = 1.08) were respectively marginally (*p* = 0.06) and significantly (*p* = 0.03) higher than the reputation of the *Generic Foreign Chinese* product (*M* = 4.37, *SD* = 1.15). Thus, H5 is confirmed (Figure 5).

**Figure 6:**
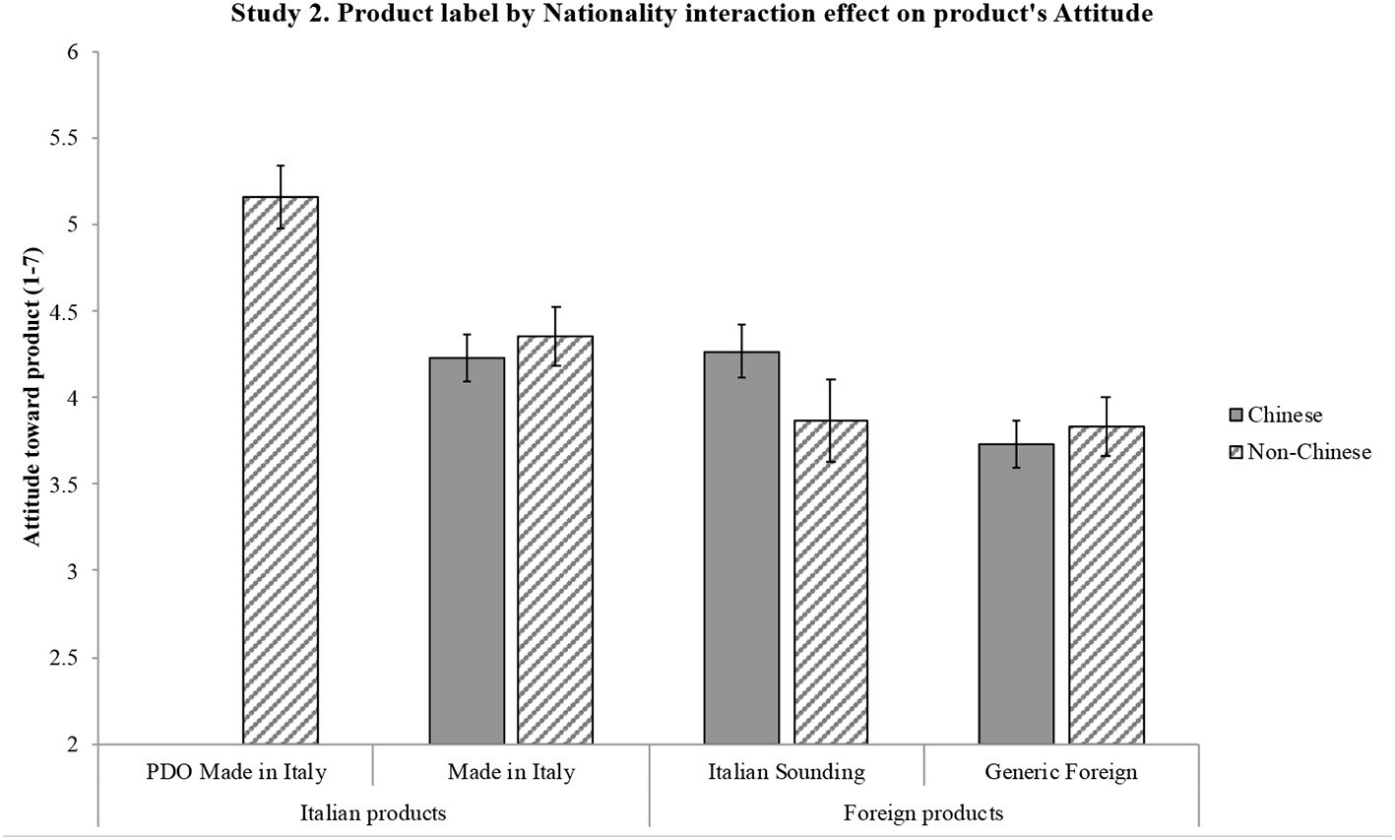
Interaction effect of product label and nationality on attitude toward product. Attitude toward product is measured on a scale from 1 (negative attitude) to 7 (positive attitude). Error bars represent 95% confidence interval of the mean. All significant differences have a *p* < 0.001. For visualization purposes, all means from the two sub-samples are plotted together; yet, pairwise comparisons are made across products within the same sample.

To test H6, the reliability of the attitude scale was sufficient or good for all levels of measurement within the subjects (*Made in Italy*, α = 0.66; *Italian Sounding*, α = 0.75; *Generic Foreign Chinese*, α = 0.65). A repeated-measures ANOVA on attitude was run, showing a significant omnibus effect of product on attitude, *F*_(2, 198)_ = 21.82, *p* < 0.001, η*p*^2^ = 0.18. The subsequent *post hoc* comparisons show that both *Made in Italy* (*M* = 4.23, *SD* = 0.71) and *Italian Sounding* (*M* = 4.27, *SD* = 0.75) products get similar scores on the attitude scale, which are both significantly higher (both *p* < 0.001) than the *Generic Foreign Chinese* product score (*M* = 3.73, *SD* = 0.71). Thus, H6 is confirmed (Figure 6).

**Figure 7:**
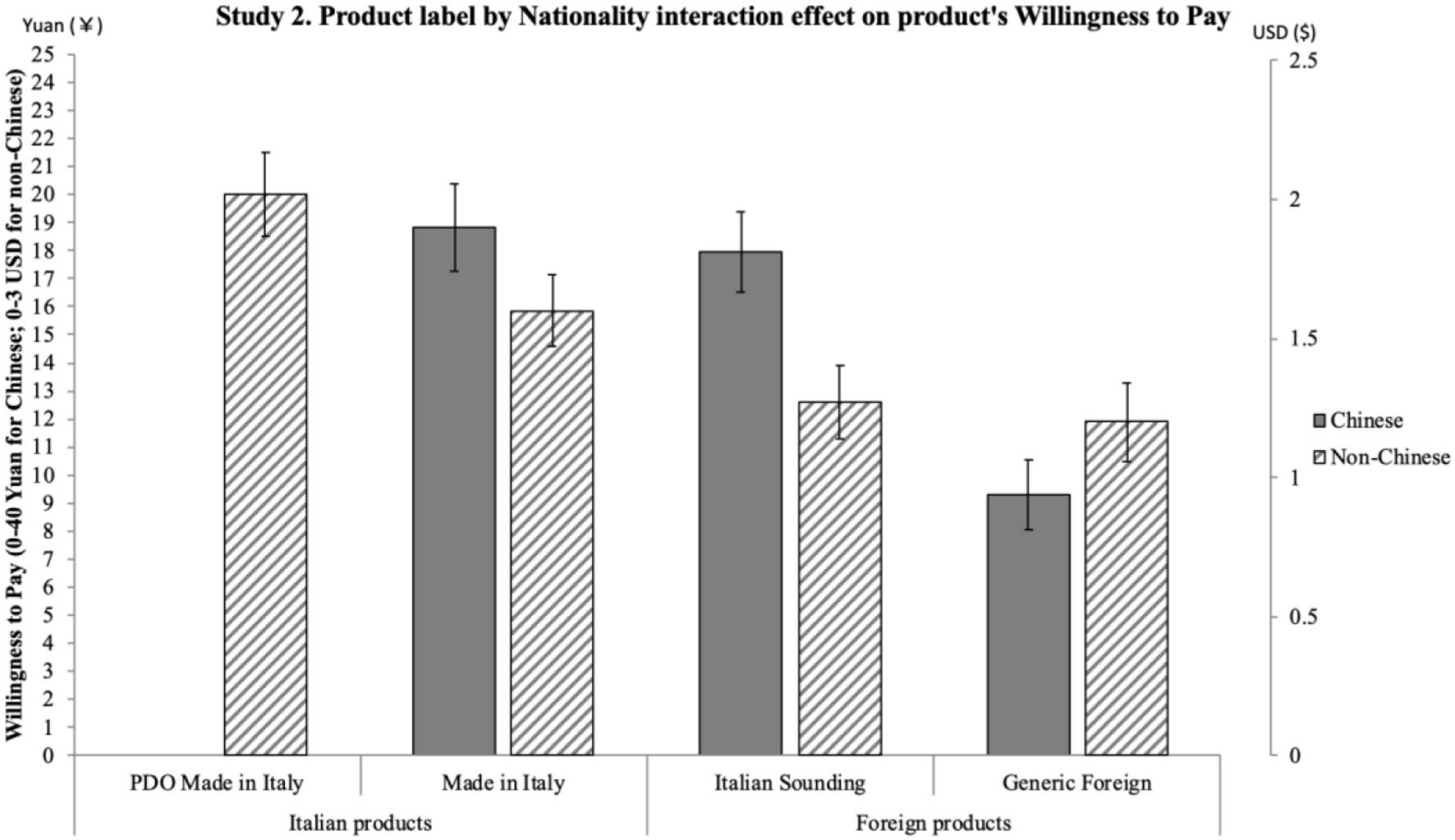
Interaction effect of product label and nationality on WTP. WTP for the product is measured from ¥0 to ¥40 for the Chinese sample (left *Y* axis) and from $0 to $3 for the non-Chinese sample (right *Y* axis). Error bars represent 95% confidence interval of the mean. All significant differences have a *p* < 0.001. For visualization purposes, all means from the two sub-samples are plotted together; yet, pairwise comparisons are made across products within the same sample.

To test H7, a repeated-measures ANOVA of product on WTP (price expressed in yuan) was conducted, showing a significant omnibus effect, *F*_(2, 198)_ = 101.56, *p* < 0.001, η*p*^2^ = 0.51. The subsequent *post hoc* comparisons show that the average price payable for *Made in Italy* (*M* = 18.84; *SD* = 7.82) and *Italian Sounding* (*M* = 17.96; *SD* = 7.18) products are similar and they are both significantly higher (both *p* < 0.001) than the *Generic Foreign Chinese* product price (*M* = 9.30; *SD* = 6.38). Thus, H7 is confirmed (Figure 7).

Regarding the second sample (non-Chinese expatriates), to test H5, a repeated-measures ANOVA confirms the hypothesis of different general reputation scores across the four products *F*_(3, 270)_ = 43.33, *p* < 0.001, η*p*^2^ = 0.32, showing that the *PDO Made in Italy* product receives a significantly higher reputation score (*M* = 5.58, *SD* = 1.11) than the *Made in Italy* one (*M* = 4.42, *SD* = 1.39, *p* < 0.001), which, in turn, received a significantly higher score than both the *Italian Sounding* (*M* = 3.77, *SD* = 1.43, *p* = 0.001) and the *Generic Foreign Chinese* (*M* = 4.00, *SD* = 1.45, *p* = 0.02). The *Italian Sounding* and the *Generic Foreign Chinese* products do not differ among them (*p* = 0.18). Thus, H5 is confirmed (Figure 5).

To test H6, the reliability of the attitude scale was good reliability for all levels of measurement within the subjects (*PDO Made in Italy*, α = 0.76; *Made in Italy*, α = 0.80; *Italian Sounding*, α = 0.89; *Generic Foreign Chinese*, α = 0.68). A repeated-measures ANOVA of products on attitude show a significant omnibus effect, *F*_(2.44, 220.06)_ = 45.70, *p* < 0.001, η*p*^2^ = 0.34. The subsequent *post hoc* comparisons show that the *PDO Made in Italy* product (*M* = 5.16, *SD* = 0.83) gets a significantly higher attitude score than the *Made in Italy* one (*M* = 4.36, *SD* = 0.85, *p* < 0.001), which, in turn, received a significantly higher attitude score than both the *Italian Sounding* product (*M* = 3.87, *SD* = 1.13, *p* = 0.001) and the *Generic Foreign Chinese* product (*M* = 3.73, *SD* = 0.76, *p* < 0.001). The *Italian Sounding* and the *Generic Foreign Chinese* products do not differ among them (*p* = 0.18). Thus, H6 is confirmed (Figure 6).

To test H7, a repeated-measures ANOVA of products on WTP (expressed in USD) was conducted, showing a significant omnibus effect, *F*_(2.62, 235.84)_ = 41.78, *p* < 0.001, η*p*^2^ = 0.32. The subsequent *post hoc* comparisons show that the average price payable for the *PDO Made in Italy* product (*M* = 2.02, *SD* = 0.69) is significantly higher than the price for *Made in Italy* product (*M* = 1.60; *SD* = 0.62, *p* < 0.001), which, in turn, was higher than the prices both for *Italian Sounding* product (*M* = 1.27; *SD* = 0.64; *p* < 0.001) and for *Generic Foreign Chinese* product (*M* = 1.20; *SD* = 0.63, *p* < 0.001). The *Italian Sounding* and the *Generic Foreign Chinese* products do not differ among them (*p* = 0.30). Thus, H7 is confirmed (Figure 7).

### Auxiliary Analysis

**Figure 8:**
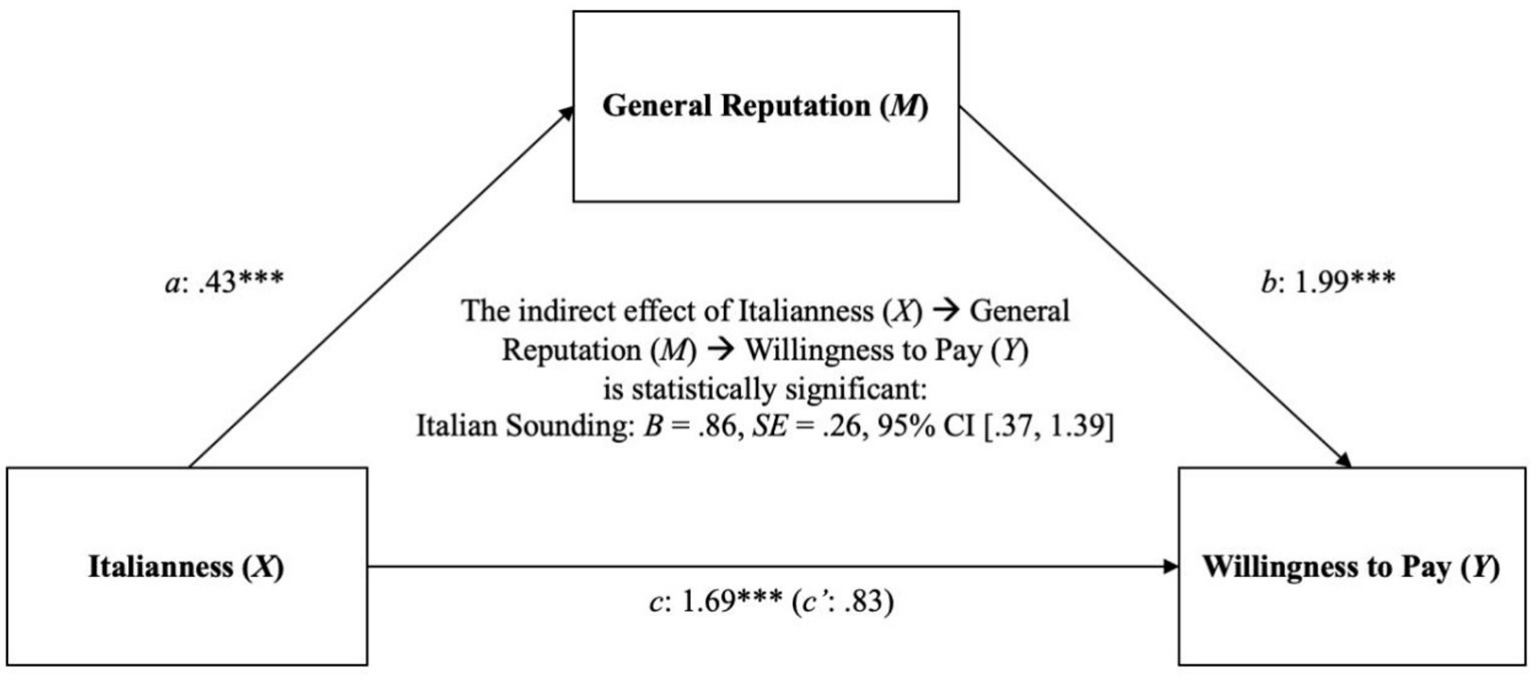
Indirect effects in Study 2 (Chinese and non-Italian expatriate residents in China) of total Italianness (X) on WTP (Y) through General Reputation (M) using bias-correcting bootstrapping (resampled 10,000 times) for each of the Italian Sounding product. When modeling the relationship between Italianness (X) and WTP (Y), total effects are shown outside parentheses and direct effects are displayed inside parentheses. ^***^p < 0.001.

Given the significant product form or label effect for WTP, an exploratory mediation analysis was conducted to corroborate Study 1's findings, by testing the indirect effect of Italianness on WTP, mediated by general reputation, only for the *Italian Sounding* product in the whole sample. The total Italianness score was computed by averaging the Italianness intensity and Italianness probability scores. The PROCESS Macro for SPSS (Model 4) was used in these analyses (Hayes, 2012). Results (Figure 8) show that the overall model was statistically significant [*R*^2^ = 0.37, *F*_(2, 188)_ = 14.61, *p* < 0.001], supporting the mediation interpretation: total Italianness increased general reputation (*b* = 0.43, 95% CI: 0.34, 0.53, *p* < 0.001), which was associated with an increase in WTP (*b* = 1.99, 95% CI: 0.82, 3.16, *p* < 0.001). The indirect effect of total Italianness on WTP via general reputation was significant (*b* = 0.87, 95% CI: 0.37, 1.39). These results suggest that the more an Italian Sounding product is perceived to be Italian, the more its reputation will increase, which, in turn, will increase consumers' WTP for that product (in ¥ for the Chinese sample, in $ for the expatriates in China sample).

### Discussion

The main aim of Study 2 is to generalize Study 1's findings by investigating how non-Italian subjects perceive, in terms first of reputation and then of attitude, and how they finally are willing to pay for a food product associated to Italy (pasta) as presented in three different forms differentiated by labeling (*Made in Italy, Italian Sounding*, and *Generic Foreign Chinese*), in China. The same hypotheses are tested, within the same Country (China) for the same product type, via two samples: first on a Chinese sample and second on a non-Italian expatriate sample (with the presence of a fourth product, *PDO Made in Italy*).

The first sample's results, consistent with expectations, basically confirm all hypotheses (H4, H5, H6, and H7), indicating that Chinese consumers do not distinguish between authentic *Made in Italy* and *Italian Sounding* products: as shown by the analyses, the scores of these two product forms almost never differ. First of all, the general reputation significantly (for the *Italian Sounding*) or with a strong tendency (for the *Made in Italy*) differs from the *Generic Foreign Chinese* product (H5); moreover, when studying the details of the specific reputation profile resulting from the 23 FRM features, this does indeed show significant differences among those three products in the detailed profile (H4). Significant differences did not emerge for *Essence*, which has to do with the more basic features of a food item. However, differences emerge for more symbolic food features such as specifically for *Cultural, Economic, Environmental*, and *Psychological Effects*, indicating that, though they did not show an advantage on the general reputation measure (compared to the *Generic Foreign Chinese* product), the *Made in Italy* product and the *Italian Sounding* product possess a very specific significantly higher reputation in certain features, which therefore allow one to understand what “Italianness” is made of, and to what extent the *Italian Sounding* can be assimilated to the *Made in Italy* in the eyes of the Chinese food consumer. As far as *Innovativeness* (*Cultural Effects*), *Context* (*Economic Effects*), *Traceability* (*Environmental Effects*), and both *Organoleptic perception* and *Conviviality* (*Psychological Effects*) are concerned, the *Made in Italy* and the *Italian Sounding* are more positively reputed than the *Generic Foreign Chinese* product. Moreover, the *Made in Italy* only (but not the *Italian Sounding*) product is more reputed than the *Generic Foreign Chinese* both for *Territorial identity* and for *Tradition* (*Cultural Effects*), while the *Italian Sounding* is more reputed than the other two products for *Lightness* and less reputed than the *Generic Foreign Chinese* for *Ability to satisfy* (both *Physical Effects*). Consequentially and coherently with the reputation profile endowing several advantages to the *Made in Italy* product, and to a slightly lesser or different extent to the *Italian Sounding* product too, subjects show an equally more favorable attitude toward both the *Made in Italy* and the *Italian Sounding* pasta products compared to the *Generic Foreign Chinese* one (H6). Finally, as expected, subjects are then willing to pay a higher price both for the *Made in Italy* (¥18.84) and for the *Italian Sounding* (¥17.96) pasta products, than for the *Generic Foreign Chinese* pasta product (¥9.30), thus confirming H7 (Huliyeti et al., 2008; Vianelli et al., 2012a). While both the Made in Italy and the Italian Sounding pack of pasta are aligned to the average price for the product, the Generic Foreign Chinese pasta pack is paid about −50% than the average product price.

The second sample's results, consistent with expectations, entirely confirm the hypotheses (H4, H5, H6, and H7), indicating that non-Italian expatriate consumers in China distinguish between authentic *PDO Made in Italy* and *Made in Italy*, and even more they distinguish among those first two compared to the *Italian Sounding* and *Generic Foreign Chinese* products. First of all, confirming the expectations, the general reputation is highest for the *PDO Made in Italy*, followed by the *Made in Italy*, while the *Italian Sounding* and *Generic Foreign Chinese* products do not differ among themselves by both having a comparatively lower general reputation (H5 confirmed). When studying more in detail the specific reputation profile resulting from the 23 FRM features, this does indeed differ among those four products (H4 confirmed). The pattern of results is pretty constant in depicting a reputation profile at the top for the *PDO Made in Italy* product, followed by the *Made in Italy* in an intermediate position, with the lowest rank occupied by both the *Italian Sounding* and the *Generic Foreign Chinese* products. Such a pattern appears across all the six areas of the reputation profile, namely, *Essence, Cultural Effects, Economic Effects, Environmental Effects, Physiological Effects*, and *Psychological Effects* (Bonaiuto et al., 2017; De Dominicis et al., 2020; as measured via the 23 features of the FRM model). There are some features, however, where the *PDO Made in Italy* and the *Made in Italy* are not significantly differentiated, as they both enjoy the same higher score of reputation for *Life time* and *Recognition* (in *Essence*); *Tradition, Familiarity*, and *Innovativeness* (in *Cultural Effects*); *Preparation* (*Economic Effects*); and *Ability to satisfy* and *Digestibility* (in *Physiological Effects*). In all other features, the *PDO Made in Italy* reputation is higher than the *Made in Italy* one, namely, *Composition* and *Genuineness* (in *Essence*); *Territorial identity* (in *Cultural Effects*); *Context* and *Price* (in *Economic Effects*); *Traceability and Safety*; as well as *Social and environmental responsibility* although with a tendency (in *Environmental Effects*); *Lightness* (in *Physiological Effects*); and *Organoleptic perception, Personal memories, Psycho-physical well-being, Conviviality*, and *Group belongingness* (in *Psychological Effects*). As for *Preparation* (in *Economic Effects*), it is the only reputation feature where the *Generic Foreign Chinese* product results with a higher score compared to both *Made in Italy* products.

Consequentially and coherently with the reputation profile endowing several advantages to the *PDO Made in Italy* product, and partly to the *Made in Italy* product, subjects show an equally more favorable attitude toward both the *PDO Made in Italy* pasta product above all, and secondly toward the *Made in Italy* one, when compared to both the *Italian Sounding* pasta product and the *Generic Foreign Chinese* one, which do differ among them (H6 confirmed). Finally, as expected, subjects are then willing to pay a higher price for the *PDO Made in Italy* pasta product ($2.02) and then for a *Made in Italy* one ($1.60), compared to both the *Italian Sounding* ($1.27) pasta product and the *Generic Foreign Chinese* pasta product ($1.20), thus confirming H7 (Huliyeti et al., 2008; Vianelli et al., 2012a). Therefore, on average, the non-Chinese expat consumer is keen to pay less than the given average price, at that time in China, for a pack of Generic Foreign Chinese pasta (about −15%) and for a pack of Italian Sounding pasta (about −20%), while affording an extra of about +7% for a Made in Italy pack of pasta and an extra of about +33% for a PDO Made in Italy pack of pasta.

Finally, the auxiliary analysis provided critical insights into the process by which a given product, when it is perceived to be Italian, might gain a financial competitive advantage over other products in China by both local citizens and expatriates: again, the more a food product is perceived to be Italian, the more positive its reputation is; in turn, the higher its reputation, the more its consumers are willing to pay for that given product. Study 3 has been subsequently planned to generalize Study 2's results to a different, equally important area of the global market.

## Study 3

### Aim and Hypotheses

As in Study 2, the main aim of Study 3 is to investigate how non-Italian subjects perceive, in terms of reputation and attitude, and are willing to pay for a food product associated to Italy (pasta) presented in three different forms (*Made in Italy, Italian Sounding*, and *Generic Foreign US*), by testing the same already confirmed hypotheses from Studies 1 and 2 on a sample of US citizens (Cembalo et al., 2008; Vianelli and Marzano, 2013).

The same hypotheses of Study 1 and Study 2 were here targeted again. It is thus expected that:

*H8:* The product form or label has an effect on the reputation profile: In particular, reputation profiles measured via the FRM are positively different for *Made in Italy* and *Italian Sounding* products compared to *Generic Foreign* products.
*H9:* The product has an effect on general reputation: In particular, general reputation is more positive for *Made in Italy* and *Italian Sounding* products compared to *Generic Foreign* products.
*H10:* The product has an effect on attitude: In particular, the attitude is more positive for the *Made in Italy* and *Italian Sounding* products compared to *Generic Foreign* products.
*H11:* The product has an effect on WTP: In particular, WTP is higher for the *Made in Italy* and *Italian Sounding* products compared to *Generic Foreign* products.

### Method

#### Participants, Procedure, and Materials

Data were collected on a sample of 237 subjects (*M* = 134; *F* = 103) having both US nationality and residence, whose age ranged from 19 to 69. The questionnaire was administered electronically in October–November 2016 in the United States via *M-Turk*, a well-known online sorting program.

In order to select the three different products, the same pre-test as Study 1 and Study 2 was used (asking a preliminary sample of about 20 persons at Claremont Graduate University to indicate two pieces of information: the most known and the most consumed Italian food product), showing that the best known and most consumed Italian product in the USA is pasta, as in China. The three selected products were (again, avoiding major brands among those actually on sell in the market at that moment): “Spaghetti Divella” as *Made in Italy*, “Spaghetti Ronzoni” as *Italian Sounding*, and “Spaghetti Anthonys” as *Generic Foreign US* (Appendix D).

#### Measures

The questionnaire, similar to the one used in Study 2, investigated three product forms (*Made in Italy, Italian Sounding*, and *Generic Foreign US*), and it was administered in American English. The whole survey is available in the Supplementary Material of this manuscript.

Similarly to Studies 1 and 2, Italianness intensity and probability were used to measure Italianness perception. To measure the product's general reputation, the same seven-point Likert-type scale item as Studies 1 and 2 was used. Food reputation profiles of the three products were measured via the same 23 items on a seven-point Likert-type scale (from “strongly disagree” to “strongly agree”), derived from FRM (Bonaiuto et al., 2017; De Dominicis et al., 2020), as those used in Study 2.

The same 10 seven-point evaluative semantic differential scales as Studies 1 and 2 were used to investigate attitude. To measure WTP, the same one-item 11-point Likert-type scale (adapted from Hanemann, 1984) as Studies 1 and 2 was used, expressing prices in US dollars (from “$0” to “$3”), where the scale's middle point (“$1.50”) was close to a possible national average price for the product in that period (USD 0.30 cumulative increase in each step). The item was: “Considering that the average price of a 16 oz (1 lb) package pasta is about $1.50, how much would you be willing to pay if you would buy 16 oz (1 lb) of PRODUCT NAME.”

As in Studies 1 and 2, all statistical analyses were released using the SPSS version 27 software.

#### Manipulation Check

To verify that the products were perceived differently as for their level of Italianness, a manipulation check was carried out on the Italianness intensity and probability to compare the three products.

Two repeated-measures ANOVAs were performed to test the effects of product label on the dependent variable “Italianness intensity” (score 0–10) and “Italianness probability” (0–100%). The manipulation checks indicated an effect of the product label both on Italianness intensity [*F*_(1.76, 351.58)_ = 222.92, *p* < 0.001, η*p*^2^ = 0.53] and on Italianness probability [*F*_(1.56, 305.22)_ = 307.69, *p* < 0.001, η*p*^2^ = 0.61], such that Made in Italy gets significantly higher scores in both Italianness intensity (*p* < 0.001) and probability (*p* < 0.001) than the Italian Sounding product, which, in turn, gets significantly higher scores in both Italianness intensity (*p* < 0.001) and probability (*p* < 0.001) than the Generic Foreign product (Table 8).

**Table 8:** Mean scores and SD of product label related to Italianness intensity and probability in US citizens (Study 3).

**Product label**	**Italianness intensity**	**Italianness probability**
	**M (SD)**	**M (SD)**
	**(*N* = 201)**	**(*N* = 196)**
Made in Italy	7.83 (1.84)	4.00 (0.98)
Italian sounding	4.47 (2.63)	2.14 (1.09)
Generic foreign	3.62 (2.56)	1.70 (0.99)

### Results

#### Comparison of Indicators of Food Reputation Across Products (H8)

As in Study 2, the results of the different repeated-measures ANOVAs were grouped in the six areas identified by the synthetic indicators of FRM (see Table 9).

**Table 9:** Mean (*M*) and Standard Deviation (*SD*) scores for 23 reputation features related to the three product labels and the relevant *p*-value indicating the statistical significance of each difference (ANOVA), for the US citizens in USA.

	**Omnibus effect**	**M (SD)**			**Significance (*****p*****-value)**		
	***F(df)*** **ηp**^**2**^	**Made in Italy** **(MI)**	**Italian sounding** **(IS)**	**Generic Foreign USA** **(GFUS)**	**MI-IS**	**MI-GFUS**	**IS-GFUS**
**ESSENCE**							
Composition	19.08 (2, 400)^***^ **0.09**	4.82 (1.15)	4.75 (1.32)	4.33 (1.32)	1.000	0.000^***^	0.000^***^
Genuineness	48.78 (1.93, 386.71)^***^ **0.20**	5.23 (1.18)	4.78 (1.34)	4.22 (1.37)	0.000^***^	0.000^***^	0.000^***^
Life time	8.33 (2, 400)^***^ **0.04**	5.39 (1.27)	5.38 (1.25)	5.09 (1.35)	1.000	0.001^**^	0.002^**^
Recognition	17.61 (2, 400)^***^ **0.08**	5.36 (1.23)	5.13 (1.24)	4.83 (1.33)	0.061	0.000^***^	0.001^**^
**CULTURAL EFFECTS**							
Territorial identity	34.47 (1.89, 377.55)^***^ **0.16**	5.09 (1.28)	4.30 (1.47)	4.10 (1.48)	0.000^***^	0.000^***^	0.186
Tradition	42.50 (1.89, 377.36)^***^ **0.15**	5.24 (1.22)	4.63 (1.36)	4.34 (1.34)	0.000^***^	0.000^***^	0.009^**^
Familiarity	18.37 (2, 400)^***^ **0.08**	5.24 (1.21)	4.83 (1.31)	4.65 (1.31)	0.001^**^	0.000^***^	0.173
Innovativeness	12.96 (1.94, 387.49)^***^ **0.06**	4.39 (1.52)	4.00 (1.54)	3.87 (1.47)	0.001^**^	0.000^***^	0.491
**ECONOMIC EFFECTS**							
*Context*	16.63 (1.87, 374.30)^***^ **0.08**	5.17 (1.13)	4.83 (1.33)	4.65 (1.53)	0.003^**^	0.000^***^	0.173
Price	8.53 (1.86, 372.04)^***^ **0.04**	5.25 (1.03)	5.16 (1.13)	4.91 (1.23)	0.794	0.001^**^	0.004^**^
Preparation	7.67 (2, 400)^**^ **0.04**	5.76 (1.02)	5.28 (1.13)	5.48 (1.27)	0.034^*^	0.001^**^	0.440
**ENVIRONMENTAL EFFECTS**							
Social and environmental responsibility	24.01 (2, 400)^***^ **0.11**	4.84 (1.09)	4.53 (1.13)	4.27 (1.09)	0.000^***^	0.000^***^	0.008^**^
Traceability	31.81 (1.94, 388.13)^***^ **0.14**	5.06 (1.20)	4.46 (1.31)	4.22 (1.37)	0.000^***^	0.000^***^	**0.055**^**∧**^
Proximity	0.623 (1.69, 337.45) 0.00	4.34 (1.50)	4.34 (1.34)	4.23 (1.34)	1.000	1.000	0.676
Safety	10.92 (1.94, 387.91)^***^	5.06 (1.11)	5.00 (1.14)	4.67 (1.30)	1.000	0.000^***^	0.001^**^
**PHYSIOLOGICAL EFFECTS**							
Ability to satisfy	6.59 (1.94, 387.54)^**^ **0.03**	5.76 (1.12)	5.67 (1.09)	5.50 (1.20)	0.781	0.000^***^	**0.070**^**∧**^
Digestibility	10.75 (2, 400)^***^ **0.05**	4.99 (1.26)	4.87 (1.42)	4.62 (1.37)	0.473	0.000^***^	0.009^**^
Lightness	9.67 (1.94, 388.26)^***^ **0.05**	3.95 (1.48)	4.17 (1.52)	3.78 (1.37)	**0.062**^**∧**^	0.112	0.000^***^
**PSYCHOLOGICAL EFFECTS**							
Organoleptic perception	22.22 (2, 400)^***^ **0.10**	5.62 (1.10)	5.38 (1.25)	5.04 (1.28)	0.022^*^	0.000^***^	0.000^***^
Personal memories	15.47 (1.80, 360.59)^***^ **0.07**	5.10 (1.41)	4.93 (1.48)	4.59 (1.42)	0.299	0.000^***^	0.001^**^
Psycho-physical well-being	9.93 (1.92, 385.09)^***^ **0.05**	4.24 (1.39)	4.16 (1.47)	3.89 (1.40)	1.000	0.000^***^	0.002^**^
Conviviality	10.36 (2, 400)^***^ **0.05**	5.47 (1.18)	5.24 (1.28)	5.09 (1.32)	0.025^*^	0.000^***^	0.231
Group belongingness	12.91 (2, 400)^***^ **0.06**	4.95 (1.12)	4.84 (1.33)	4.49 (1.37)	0.753	0.000^***^	0.001^**^

*Bold values represents significant or marginally significant effects*.

∧ *p < 0.10*;

* *p < 0.05*;

** *p < 0.01*;

*** *p < 0.001*.

As for the Essence scores, results show that all dimensions contribute to defining the distinctive reputation profiles of the products. In particular, for Composition, Life time, and Recognition, the results of the Made in Italy and Italian Sounding products do not significantly differ among them, although both scored higher reputation levels compared to the Generic Foreign US product. As regards Genuineness, the Made in Italy product significantly differs from both other product forms; however, the Italian Sounding product has still higher scores compared to the Generic Foreign US product.

As regards *Cultural Effects*, results for *Territorial identity, Familiarity*, and *Innovativeness* show a similar trend: namely, the *Generic Foreign US* and the *Italian Sounding* products do not significantly differ, while the *Made in Italy* product records higher scores in all dimensions. Only for *Tradition* does the *Italian Sounding* product have a more positive reputation than the *Generic Foreign US* product, and the *Made in Italy* confirms its excellence here too.

As regards Economic Effects, the Made in Italy product significantly (or by a strong tendency) excels with respect to the other two products, both for Context and for Preparation. However, the Italian Sounding product succeeds in marking a positive reputation significantly different with respect to the Generic Foreign US product for Price, equalling the Made in Italy product.

As regards *Environmental Effects*, apart from Proximity, which does not differentiate the three products, the three dimensions *Social and environmental responsibility, Traceability*, and *Safety* all show the significant (or strong tendency) reputation advantage of both *Made in Italy* and *Italian Sounding* products compared to the *Generic Foreign US* product, while the *Made in Italy* product positively differentiates itself from the *Italian Sounding* one only in the first two features, not in the last one above.

As for *Physiological Effects*, again both *Made in Italy* and *Italian Sounding* products get an equally positive reputation profile, which is significantly (or with a strong tendency) higher than the *Generic Foreign US* product in both *Ability to satisfy* and *Digestibility* dimensions, while in the *Lightness* dimension, the *Italian Sounding* product enjoys the best reputation among all products.

As for *Psychological Effects*, the mean score of the *Made in Italy* product is always significantly higher than the *Generic Foreign US* one, and the Italian Sounding product follows the same pattern of results (whether with significance or a strong tendency), for all features except *Conviviality*, namely, *Organoleptic perception, Personal memories, Psycho-Physical Well-being*, and *Group belongingness*. *Organoleptic perception* and *Conviviality* are the only two features where the *Made in Italy* product manages to significantly differentiate its reputation profile from the *Italian Sounding* product.

#### Comparison of General Reputation, Attitude, and WTP Across Products (H9–H10–H11)

**Figure 9:**
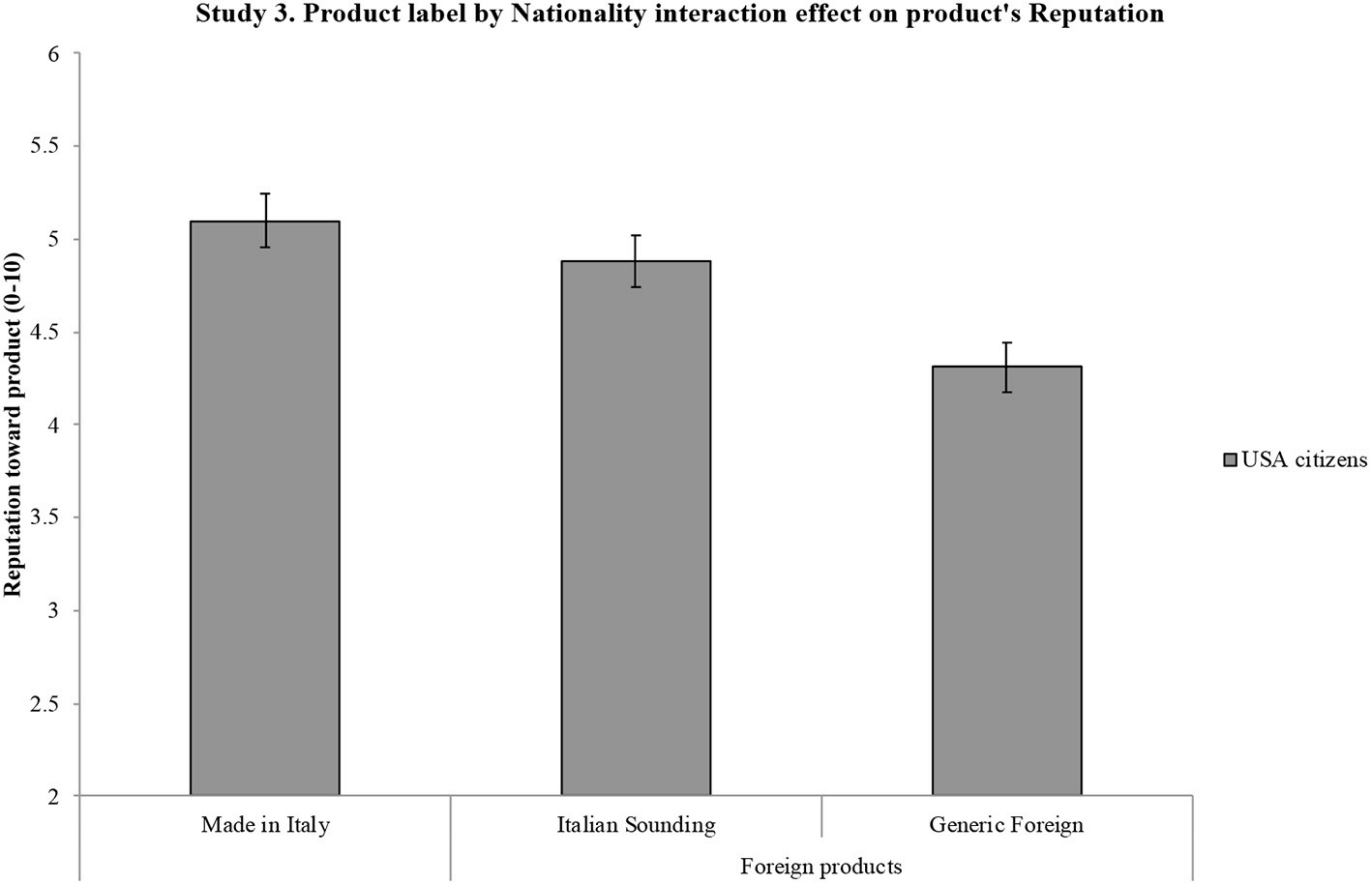
Interaction effect of product label and nationality on product's reputation. Reputation toward product is measured on a scale from 1 (negative reputation) to 10 (positive reputation). Error bars represent 95% confidence interval of the mean. All significant differences have a *p* < 0.001.

To test H9, a repeated-measures ANOVA of products on the general reputation scores was conducted, showing a significant omnibus effect, *F*_(2, 400)_ = 45.72, *p* < 0.001, η*p*^2^ = 0.19. The subsequent *post hoc* comparisons show that the reputation mean scores of *Made in Italy* product (*M* = 5.10, *SD* = 1.00) were significantly higher than the reputation score of the *Italian Sounding* product (*M* = 4.88, *SD* = 1.00, *p* < 0.001), which, in turn, was significantly higher than the reputation score of the *Generic Foreign US* product (*M* = 4.31, *SD* = 0.93, *p* < 0.001) (H9 confirmed; Figure 9).

**Figure 10:**
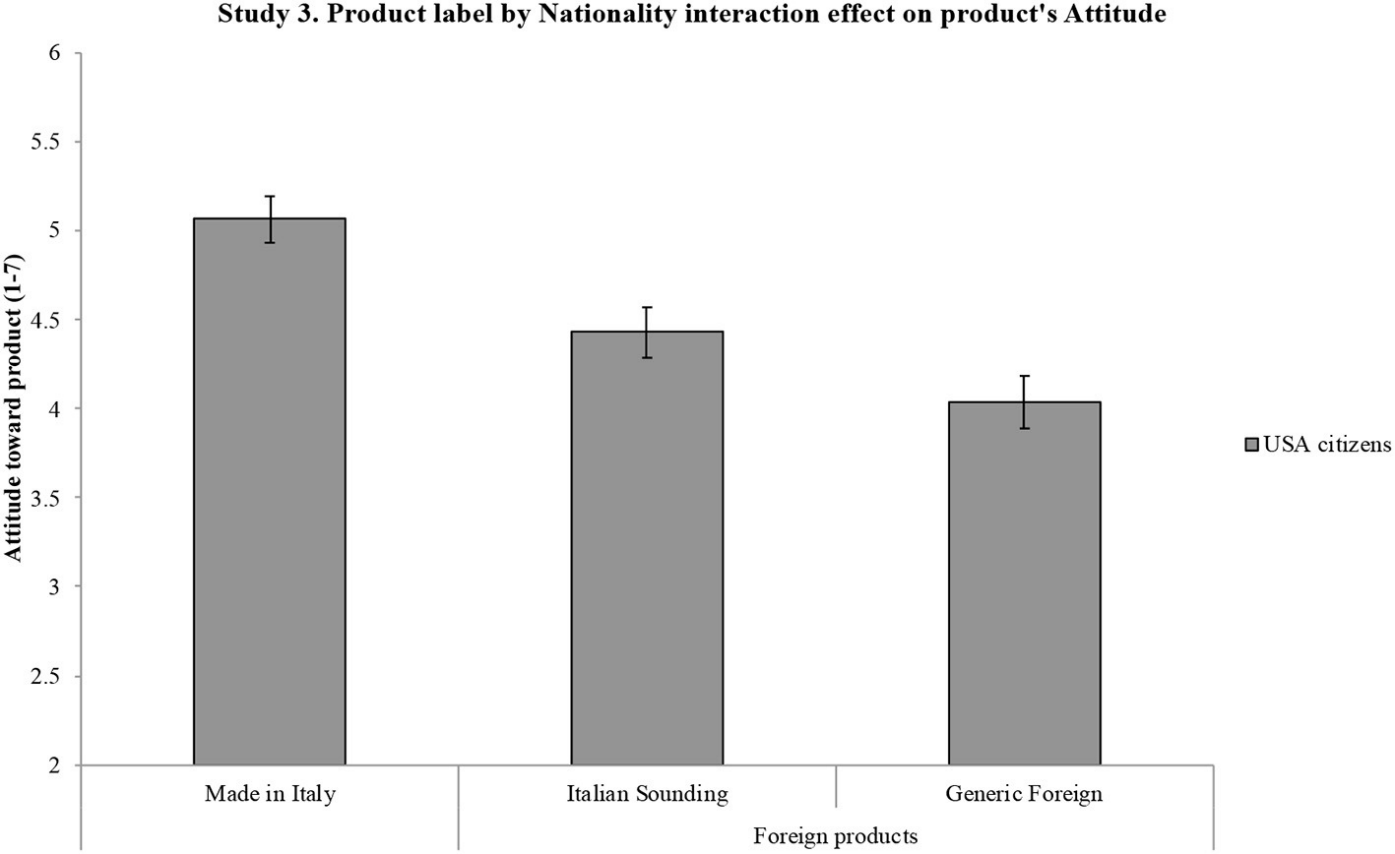
Interaction effect of product label and nationality on attitude toward product. Attitude toward product is measured on a scale from 1 (negative attitude) to 7 (positive attitude). Error bars represent 95% confidence interval of the mean. All significant differences have a *p* < 0.001.

To test H10, the reliability of the attitude scale was excellent for all levels of measurement within the subjects (*Made in Italy*, α = 0.87; *Italian Sounding*, α = 0.89; *Generic Foreign US*, α = 0.89). A repeated-measures ANOVA on attitude was run, showing a significant omnibus effect of product on attitude, *F*_(1.92, 383.79)_ = 81.93, *p* < 0.001, η*p*^2^ = 0.29. The subsequent *post hoc* comparisons show that attitude toward *Made in Italy* product (*M* = 5.07, *SD* = 0.91) was significantly higher than the attitude toward the *Italian Sounding* product (*M* = 4.43, *SD* = 0.98), which, in turn, was significantly higher than the attitude toward the *Generic Foreign US* product (*M* = 4.03, *SD* = 1.00) (H10 confirmed; Figure 10).

**Figure 11:**
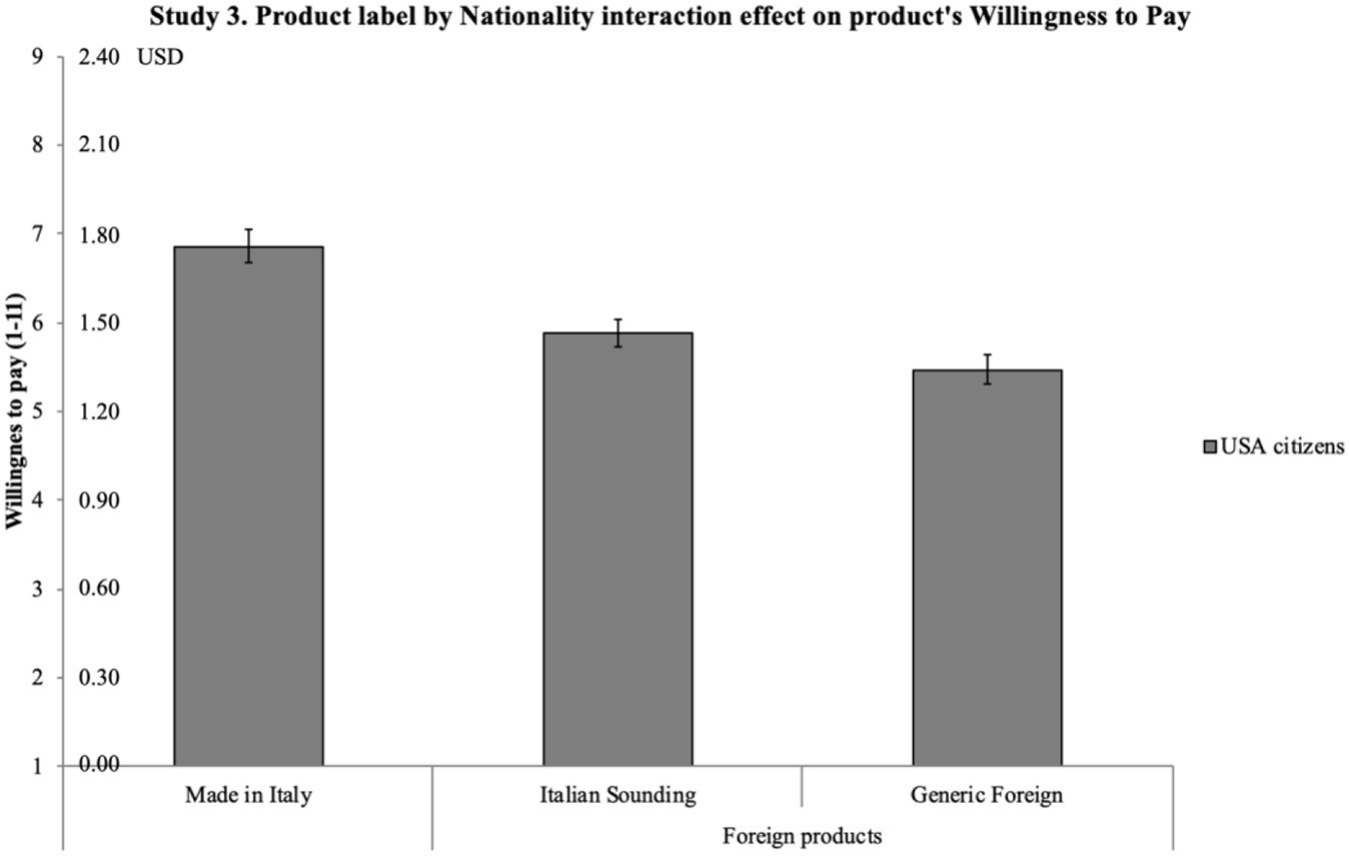
Interaction effect of product label and nationality on WTP. WTP for the product is measured from 1 (WTP a lower price) to 11 (WTP a higher price). WTP ranged from $0.00 to $3.00 (with one step increase in the Likert scale corresponding to an increase of $0.30). Error bars represent 95% confidence interval of the mean. All significant differences have a *p* < 0.001.

To test H11, a repeated-measures ANOVA of product on WTP (price expressed in USD) was conducted, showing a significant omnibus effect, *F*_(1.74, 348.99)_ = 92.05, *p* < 0.001, η*p*^2^ = 0.31. The subsequent *post hoc* comparisons show that the average price payable for *Made in Italy* (*M* = 6.88; *SD* = 1.49) was significantly higher than the price for the *Italian Sounding* product (*M* = 5.88; *SD* = 1.10), which, in turn, was significantly higher than the price for the *Generic Foreign US* product (*M* = 5.47; *SD* = 1.22) (H11 confirmed; Figure 11).

### Auxiliary Analysis

**Figure 12:**
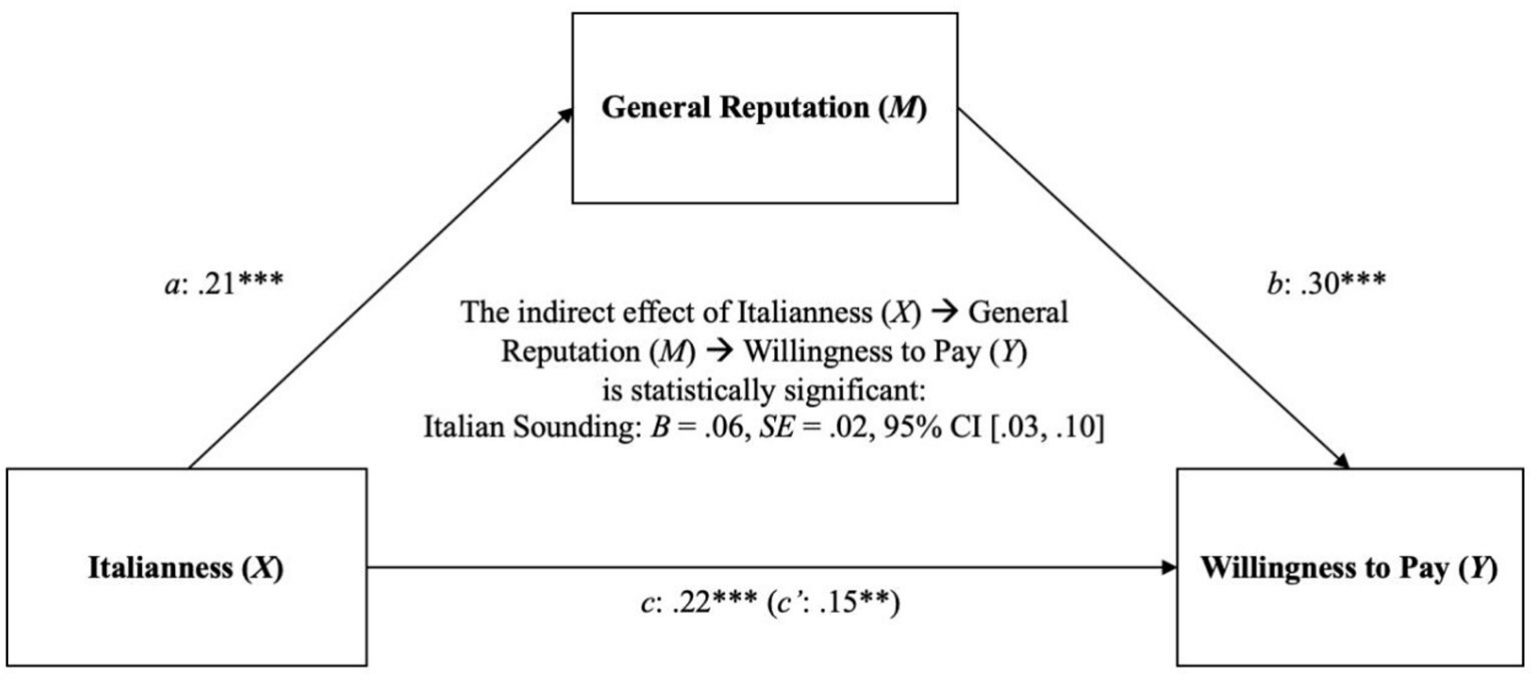
Indirect effects in Study 3 (US citizens and residents in the USA) of Italianness (X) on WTP (Y) through General Reputation (M) using bias-correcting bootstrapping (resampled 10,000 times) for each of the Italian Sounding product. When modeling the relationship between Italianness (X) and WTP (Y), total effects are shown outside parentheses and direct effects are displayed inside parentheses. ^***^p < 0.001.

Given the significant label effect for WTP, an exploratory mediation analysis was conducted to corroborate the findings of Studies 1 and 2, by testing the indirect effect of Italianness on WTP, mediated by reputation, for the Italian Sounding product in the USA. The Italianness score was computed by averaging the Italianness intensity and Italianness probability scores. The PROCESS Macro for SPSS (Model 4) was used in these analyses (Hayes, 2012). Results (Figure 12) show that the overall model was statistically significant [*R*^2^ = 0.19, *F*_(2, 194)_ = 22.65, *p* < 0.001], supporting the mediation interpretation: Italianness increased reputation (*b* = 0.21, 95% CI: 0.13, 0.28, *p* < 0.001), which was associated with an increase in WTP (*b* = 0.30, 95% CI: 0.15, 0.45, *p* < 0.001). The indirect effect of Italianness on WTP via reputation was significant (*b* = 0.06, 95% CI: 0.03, 0.10). These results suggest that the more an Italian Sounding product is perceived to be Italian, the more its reputation will increase, which, in turn, will increase consumers' WTP for that product (in $ for this US sample).

### Discussion

As expected, results fully confirm all hypotheses H8, H9, H10, and H11. US residents evaluate more positively the *Italian Sounding* product compared to the *Generic Foreign US* product. However, the most positively evaluated product is the *Made in Italy* one (Vianelli and Marzano, 2013; Pegan et al., 2014). Specifically, results show that the Italianness of the product influences the overall evaluation of the product: although the *Made in Italy* product is considered the best as for the investigated features, it is immediately followed by the *Italian Sounding* one in all analyses (H8). Specifically, the *Made in Italy* product best represents the Italian product reputation profile by becoming the leader in basically all the FRM 23 areas (in most cases with statistical significance, in a few cases with strong tendencies), except in *Lightness* where the *Italian Sounding* significantly overrides it. On the whole, the *Italian Sounding* product manages to significantly (or with a strong tendency) emulate the reputation profile of the Italian product, especially by marking a positive difference compared to the *Generic Foreign US* under many respects: *Composition, Genuineness, Life time*, and *Recognition* (i.e., all the *Essence* area features); *Tradition* only in the *Cultural effects* area; *Price* only in the *Economic Effects* area; *Social and environmental responsibility, Traceability*, and *Safety* (i.e., all the *Environmental Effects* area except one, *Proximity*); *Ability to satisfy, Digestibility*, and *Lightness* (i.e., all the *Physiological Effects* area features); and basically all the *Psychological Effects* area features in terms of significance (*Organoleptic perception, Personal memories, Psycho-physical Well-being*, and *Group belongingness*). Coherently (H9), the general reputation average score is significantly higher for the *Made in Italy* product than for the other two products, but here again the *Italian Sounding* product marks a general reputation advantage with regard to the *Generic Foreign US*. Consequently (H10), the consumer's average attitude becomes most positive toward the *Made in Italy* product, but the *Italian Sounding* product receives a more positive attitude than the *Generic Foreign US*. Finally (H11), on average, the US consumer is keen to pay just the given average price, at that time in California, for a pack of *Generic Foreign US* pasta (about $1.50), while affording about an extra +10% for an *Italian Sounding* pack of pasta (about $1.65), and an extra +30% for a *Made in Italy* pack of pasta (about $2.00).

Finally, the auxiliary analysis provided critical insights into the process by which a given product, when it is perceived to be Italian, might gain a financial competitive advantage over other products, by US citizens too: here, again, the more a food product is perceived to be Italian, the more positive its reputation is; in turn, the higher its reputation, the more its US consumers are willing to pay for that given product.

## General Discussion

This research was designed to evaluate and compare the perception of products associated with Italy and to test the effects of the product label on attitude, reputation, and WTP for different product forms, with a particular focus on IS products, compared to Made in Italy and Generic Foreign ones. Results of the three empirical studies, consistent with expectations and literature (Thakor and Lavack, 2003; Nicoletti et al., 2007; Balabanis and Diamantopoulos, 2008, 2011; Bursi et al., 2012), suggest that different cultural contexts (Italy, China, and the USA) show a different attitude and reputation toward the products differentiated by the “Italianness” of their label, as well as an impact of such product reputation on WTP (Landon and Smith, 1998; Loureiro and McCluskey, 2000; Loureiro and Hine, 2002).

We found that, in Italian and EU consumers (Study 1), the Italian Sounding product did not gain reputation, attitude toward it, or WTP for it, compared to the other products. However, in China and in the USA (Studies 2 and 3), made in Italy products had a higher reputation compared to Italian Sounding products, which, in turn, were perceived more positively by consumers and had a higher reputation compared to Generic Foreign ones. This confirms how the Italian Sounding product label, by recalling an alleged Italian identity of the product and thus increasing its attractiveness, brings benefits to producers, exploiting the high popularity of Made in Italy specialty products and their high reputation around the world, by achieving an overall better reputational judgment from consumers, who therefore develop a positive attitude toward it. This can be explained by references to Italianness on the label itself (via brand name or iconic features), encouraging customers to mistakenly associate the product with features typical of Made in Italy products (Liefeld, 2004; Balabanis and Diamantopoulos, 2008, 2011). Thus, consumers' perception of the product's reputation, their attitudes toward the product, and their WTP are all positively affected by an Italian Sounding label. Furthermore, to ascertain the process by which these effects might occur, we run a series of auxiliary mediation analyses. Our results, consistently across Studies 1, 2, and 3, suggest that the perceived “Italianness” of a product increases its perceived reputation together with endowing a premium price: thus, the more a product is perceived to be “Italian,” the more this perception increases the believed quality features applied to the product, which gains an added cost.

We further investigated this effect by decomposing the reputational profile of the tested products in different samples (Study 2 and 3), according to the Food Reputation Map model's 23 dimensions (Bonaiuto et al., 2017; De Dominicis et al., 2020). Therefore, the present contribution shows for the first time in a systematic and cross-cultural way that this reputational boost—above and beyond a general reputation halo—endows the product with a specific positive reputational profile. This result shows in detail the reputation features that are specifically boosted in the considered agro-food product (pasta) thanks to the Italian Sounding phenomena (by also showing that other specific features in the same reputational profile do not enjoy such a positive halo effect). Specifically, results show that the values transposed on the Italian Sounding product—justifying a greater expense compared to the purchase of a foreign product—are not fuzzy or undefined, or generic ones: rather, they specifically pertain mainly to psychological and physiological well-being, as well as production responsibility, ensuring safe and reliable purchase choices. Such a result highlights the specific added value of “Italianness” within the agro-food consumption sector. It should be noted that the reputational features profile—which aims to positively differentiate the Italian Sounding product from the generic foreign one (the Chinese one in China and the American one in the USA)—mimics those features that positively differentiate both the Made in Italy one and the PDO Made in Italy one from the generic foreign product. It is thus clear that the Italian Sounding label is not simply advantaging in general the product's reputation, attitude, and WTP; rather, the Italian Sounding is doing so by granting the product a higher perceived Italianness that is associated with a cluster of reputational features assimilating it to the same reputational profile of the Made in Italy products (the standard one and especially the PDO one). Within such a scenario, results also show that only very few features remain to differentiate, if any, the Made in Italy products from the Italian Sounding ones. Our results, though limited, show that they still exist: such a residual reputational capital could be considered in terms of practical implications; i.e., features of the Italian Sounding in the future could be mimicked and those of the Made in Italy could thus be defended. The few features still differentiating Made in Italy products from Italian Sounding ones could therefore be considered as leverages for the next strategies aiming to maintain a distinction between merely Italian Sounding products and truly Made in Italy ones. This effort should however be coupled with prospective plans for recovering what today seems a lost distinction over those many features presently not enjoyed by Made in Italy anymore as differentiating assets from the Italian Sounding products.

These psychological processes in turn increase consumers' WTP for that product, in terms of average yuan the Chinese consumer is willing to pay for a pack of pasta in China, and in terms of dollars the US consumer is willing to pay for a pack of pasta in the USA. By considering the average amounts resulting from the samples of Study 2 (Chinese) and Study 3 (Americans), the US consumer in the USA is ready to pay an added cost of about 7–8% for an Italian Sounding pack of pasta (compared to a corresponding American product) in the USA, while his/her Chinese counterpart, in China, is ready to pay an extra 93% more for an Italian Sounding pack of pasta (compared to a corresponding Chinese product) in China. Moreover, average WTP data also show that the average amount of yuan the Chinese consumer is willing to pay for an Italian Sounding pack of pasta is aligned to the same amount s/he is willing to pay for a real Made in Italy one.

Finally, such a pattern of Italian Sounding results appears within specific samples only: i.e., Chinese consumers in China and US consumers in the USA. On the contrary, Italian consumers and EU consumers in Italy and the EU (Study 1), as well as expatriates, that is, non-Chinese consumers in China, are immune to Italian Sounding effects. This is in line with literature stressing that the COO effect should also be considered in the light of the specific characteristics of the consumer samples (such as socio-demographics and personality, Bilkey and Ness, 1982). Our results seem to indicate that cultural and/or geographical distance may matter here. However, as the samples contrasted within a country (e.g., Chinese vs. non-Chinese expatriates in China), with differences according to several variables, it is difficult to detect which is the crucial variable, i.e., which exact variable is discriminating between people vulnerable to the Italian Sounding effects and people who are immune to the Italian Sounding phenomenon, within a given context and market. One possibility is that an Eastern vs. Western dimension plays a role here, in the sense that people from an Eastern culture and country are sensitive to the Italian Sounding effects, while people from a Western culture and country are not. This interpretation, however, does not agree with the non-Chinese expatriates in the Chinese sample's composition and results. In fact, such a sub-sample comprises both members from Western and Eastern cultures: i.e., about 42% from USA, Germany, and Switzerland; about 32% from Malaysia, Singapore, North Korea, and Iran; and a quarter of the sample from other countries. By comparing Italian Sounding effects in the various samples considered across the three studies, it is evident that Italians showed basically no vulnerability to Italian Sounding effects; EU people showed a similar pattern, with only a small, residual vulnerability to Italian Sounding effects in the sense of a higher perception of its Italianness and reputation, attitude, and WTP compared to the Italian sample; non-Chinese expatriates in China showed a similar residual vulnerability to Italian Sounding effects, in the sense of equalling its assessment to the generic foreign Chinese product available in the same domestic market; Americans showed a limited but already significant vulnerability to Italian Sounding effects (at reputational, attitudinal, and WTP levels); Chinese people showed a conspicuous vulnerability to Italian Sounding effects (at all those levels, by also reporting, among all considered samples, the greatest magnitude in terms of consumption implication outcome). The crucial differences between Chinese people and non-Chinese expatriates in China should be carefully considered in order to appreciate this issue. Thus, future research efforts could try to better focus on which variables of a sample are capable of moderating the Italian Sounding effects at perceptual, reputational, attitudinal, and decision-making levels. On the basis of our three-study results pattern, several potential candidate variables could be short-listed for such a test: geographical distance from the country of origin allegedly referred to by the IS (the greater the geographical distance from Italy, the greater the vulnerability to the IS effects); sample familiarity and knowledge familiarity with, and knowledge of the context of the target product, i.e., of the country of origin allegedly referred to by the IS (the less the experience with Italian products, the greater the vulnerability to the IS effects); and psychological distance from language or other group markers compared to an attractive target group, i.e., from the country of origin allegedly referred to by the IS (the greater the social–psychological distance from a high reputation target group, the greater the vulnerability to the IS effect). Of course, a proper test would need to measure or manipulate such moderating variables in order to statistically check their inhibiting or magnifying impacts on the effect that Italian Sounding, and its related Italianness perception, has on food reputation and, via it, on agro-food consumption attitudes and decision-making.

However, despite the clear potential of these insights in terms of their social–psychological, market, and policy-making applicability, this research presents some limitations. The methodology did not consider the priming effect in the presentation of the different product labels, so that the order of presentation might have influenced the subjects' responses. It could be possible that the non-randomization of the stimuli somehow influenced the results. However, results from auxiliary analyses (six mediation models) confirmed the hypothesis that the Italianness of a product influences consumers' WTP via an increase in its reputation. These results support the idea that the gain in reputation associated with Italian Sounding products, rather than presentation order, drives consumer assessments of that product. Of course, to exclude all possible confounding effects, a replication of our experiments where conditions will be presented in a random order should be conducted. Within the present data set, it can be stressed that—even if in principle order and sequence confounding effect cannot be excluded—such bias does not impede the emergence of the described Italian Sounding effect at reputational, attitudinal, and purchasing payment intention levels, in the different cultural, linguistic, and national samples considered. It seems relatively implausible, from a theoretical point of view, that such a methodological artifact would play a greater or exclusive role for some cultural sub-samples in interaction with some of the products only, by magnifying the resulted effects precisely in the direction of the hypothesis only (also considering the fact that most hypotheses regarded complex interaction effects).

Another methodological limit is that the WTP measure has been implemented in slightly different ways in some cases: in the Italian and EU samples, the origin value in the measurement scale of the two products is not homogeneous, as for one product it starts from zero, while for the other product, it starts from a price value that is above zero; in all subsequent studies, price origins start from zero. Therefore, future attempts should keep the WTP operationalization always constant to afford proper comparisons. At the same time, the present results' consistency, in spite of such slight methodological differences, corroborates the generalization of the WTP effect described in the present contribution.

A third methodological limitation is the use of scales translated from Italian into English and then into Chinese, rather than previously validated tools for those cultural contexts (although the 23 profile reputation dimensions have been recently cross-culturally validated by De Dominicis et al., 2020).

To conclude, hopefully the results of this research will encourage further investigations on the Italian Sounding phenomenon, with the goal of hindering its negative effects on Italian economy [OECD (Organization for Economic Cooperation Development), 2008; Canali, 2012; EURISPES, 2013; Federalimentare, 2016].

## Data Availability Statement

The raw data supporting the conclusions of this article will be made available by the authors, without undue reservation.

## Ethics Statement

Ethical review and approval was not required for the study on human participants in accordance with the local legislation and institutional requirements. Written informed consent for participation was not required for this study in accordance with the national legislation and the institutional requirements. However, written informed consent was implied via completion of the questionnaire.

## Author Contributions

FB, SD, and MB designed the research questions and the study. SD, MB, WC, and JM supervised data collection. FB and UG contributed to data collection. FB, SD, UG, and MB drafted the manuscript. WC, JM, and MB provided feedback on the manuscript. SD performed the statistical analyses. All authors provided feedback on the final version of the manuscript.

## Conflict of Interest

The authors declare that the research was conducted in the absence of any commercial or financial relationships that could be construed as a potential conflict of interest.

## Footnotes

^1^H1–H3 in Study 1, H5–H7 in Study 2, and H9–H11 in Study 3 have been tested also considering gender and age as covariates. The covariates were non-significant and therefore are not considered in the present final version.
