# Supplementary material for: Italian Food? Sounds Good! Made in Italy and Italian Sounding Effects on Food Products' Assessment by Consumers
Source: Front Psychol. 2021 Mar 3;12:581492. doi: 10.3389/fpsyg.2021.581492 (PMC7966714; doi:10.3389/fpsyg.2021.581492)
Supplement: Supplementary file 1 [file Presentation_1.pdf]

Italian food? Sounds good! Made in Italy and Italian sounding effects on food products' assessment by consumers

Supplementary Information

English version of the Questionnaire used in Study 1 – 2 – 3

SAPIENZA
UNIVERSITÀ DI ROMA

QUESTIONNAIRE

Dear Sir/Madam, thank you for your precious collaboration. We ask you to kindly fill in the attached questionnaire for the purpose of a research that studies people's opinions on certain food. The questionnaire consists of 4 pages and it will require less than 5 minutes of your time. You are simply asked to rate each statement or question as spontaneous as possible expressing your opinion, marking each time with an X the answer that best represents your opinion. The questionnaire is completely anonymous.

Reference product:
SPAGHETTI GENTILE PASTA DI GRAGNANO I.G.P.

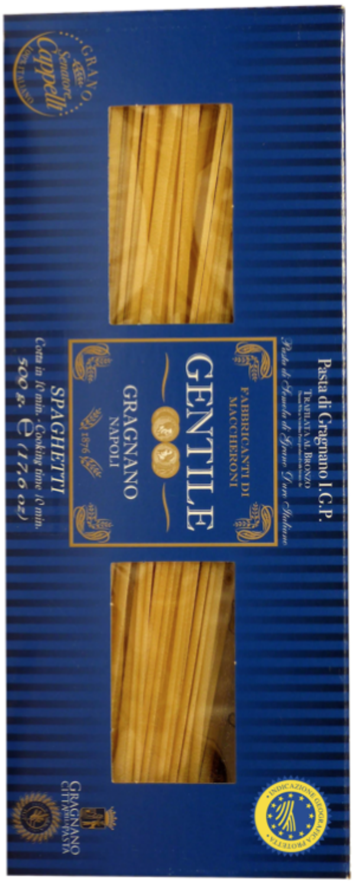

1) In the following table there are pairs of adjectives of opposite meaning. For each pair, please indicate with an X the one that best represent your opinion in relation to the product it refers to.

	Totally 1	2	3	Neither / Neither 4	5	6	Totally 7	
Bad	◻	◻	◻	◻	◻	◻	◻	Good
Counterfeit	◻	◻	◻	◻	◻	◻	◻	Authentic
Natural	◻	◻	◻	◻	◻	◻	◻	Artificial
Genuine	◻	◻	◻	◻	◻	◻	◻	Manipulated
True	◻	◻	◻	◻	◻	◻	◻	False
Undesirable	◻	◻	◻	◻	◻	◻	◻	Desirable
Without certificate	◻	◻	◻	◻	◻	◻	◻	With certificate
High quality	◻	◻	◻	◻	◻	◻	◻	Low quality
High range	◻	◻	◻	◻	◻	◻	◻	Low range
Economic	◻	◻	◻	◻	◻	◻	◻	Expensive

2) In your opinion, how much Italian is the product?

In no way | Completely

0	1	2	3	4	5	6	7	8	9	10

3) *In your opinion, how much is it likely that the product is produced in Italy?*
(0% = definitely produced abroad; 100% = definitely produced in Italy)

0%	25%	50%	75%	100%

4) In your opinion, where does the product come from? Abroad ◻ *Italy* ◻

5) Considering that the average price of a 500g of pasta is about €2.00, how much would you be willing to pay if you would buy 500g of ***SPAGHETTI GENTILE PASTA DI GRAGNANO I.G.P.*** *?*

€ 0.00	€ 0.40	€ 0.80	€ 1.20	€ 1.60	€ 2.00	€ 2.40	€ 2.80	€ 3.20	€ 3.60	€ 4.00

6) This Pasta reputation is:

Completely negative	Very negative	Fairly negative	Neither neg./ nor pos.	Fairly positive	Very Positive	Completely positive
1	2	3	4	5	6	7

7) Please answer the following questions (only for this group, don't answer if already done in the previous pages):
7a) Gender:________ 7b) Age:____ 7c) Country of origin:_____________ 7d) Country of residence:__________

THANK YOU VERY MUCH FOR YOUR PRECIOUS COLLABORATION

APPENDIX A

The four selected oil product images for Study 1

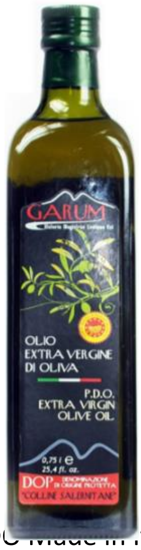

PD[illegible]taly

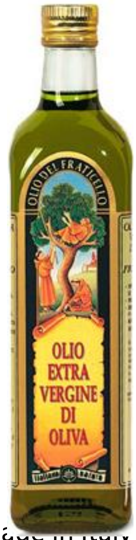

Ma[illegible]

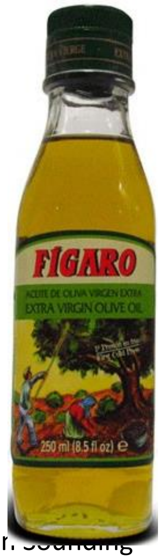

Italia[illegible]

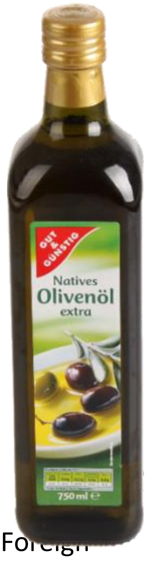

Generic Fo[illegible]

The four selected pasta product images for Study 1

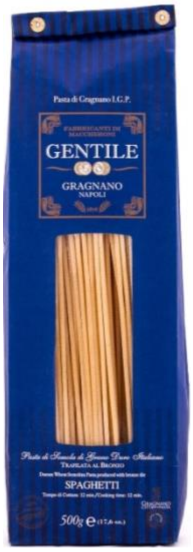

PDO Made in Italy

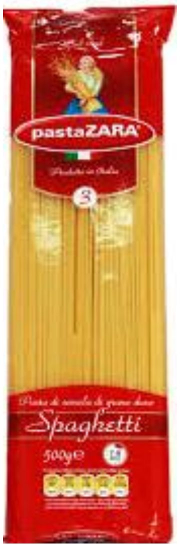

Made in Italy

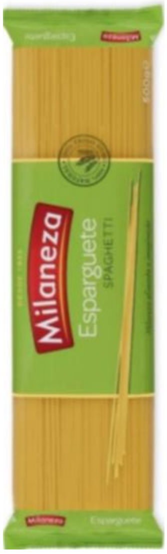

Italian Sounding

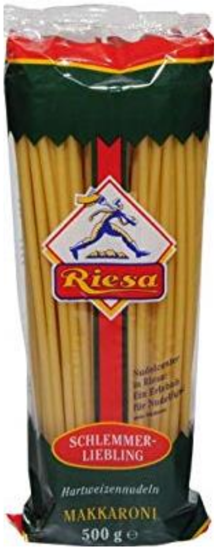

Generic Foreign

APPENDIX B

The three selected pasta product images for Study 2. In the second sample only, a fourth product was also involved, namely, an Italian PDO brand.

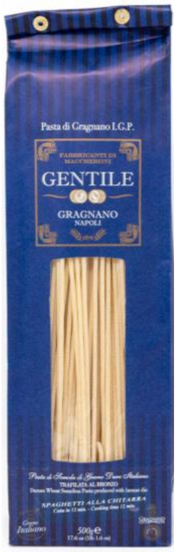

PDO Made in Italy

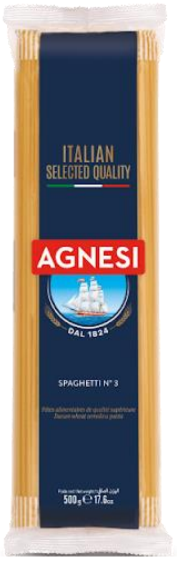

Made in Italy

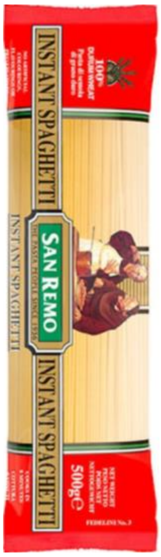

Italian Sounding

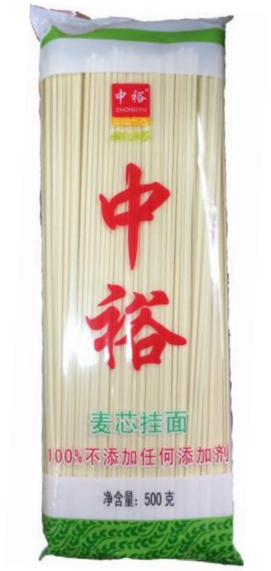

Generic Foreign Chinese

APPENDIX C

4 items measured *Essence*; 4 items measured *Cultural Effects*; 3 items measured *Economic Effects*; 4 items measured *Environmental Effects*; 3 items measured *Physiological Effects*; 5 items measured *Psychological Effects*

Synthetic Indicator	*Specific Indicator*	*Item*
ESSENCE	Composition	It has a healthy nutritious composition
	Genuineness	It is genuine and natural
	Life time	It is easy to preserve so that it does not decompose quickly
	Recognition	It is easy to identify it and its flavour
CULTURAL EFFECTS	Territorial identity	It is bonded with a territory and its local community identity
	Tradition	It relates to traditional ingredients and methods
	Familiarity	It links to personal habits and familiar taste
	Innovativeness	It is novel in taste, appearance, consistency
ECONOMIC EFFECTS	Context	It is bought or consumed in a clean, pleasant, cool place
	Price	It has a worth price/quality ratio
	Preparation	It is easy and quick to prepare
ENVIRONMENTAL EFFECTS	Social and environmental responsibility	Its production is socially and environmentally responsible and sustainable
	Traceability	Its origin is known, track-able, certified
	Proximity	It is consumed near its place of production
	Safety	It respects safety regulations and checks
PHYSIOLOGICAL EFFECTS	Ability to satisfy	It satisfies hunger and satiates
	Digestibility	It is easy to digest and light for the body
	Lightness	It contributes to physical health and it prevents diseases
PSYCHOLOGICAL EFFECTS	Organoleptic perception	It tastes good and it is appetizing
	Personal memories	It recalls childhood and past memories
	Psycho-physical wellbeing	It contributes to mental and physical wellness
	Conviviality	It is suitable to social situations and enjoyable in company
	Group belongingness	It reinforces group identity and it connects people with their own social category, group, family

APPENDIX D

The three selected pasta product images for Study 3

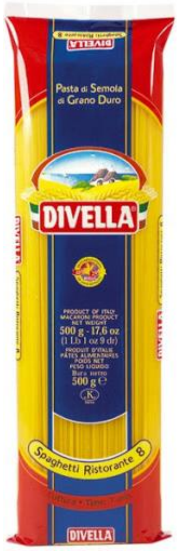

Made in Italy

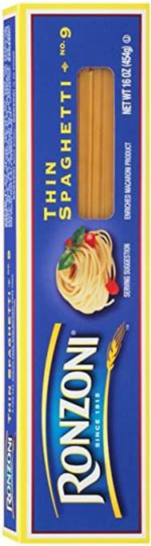

Italian Sounding

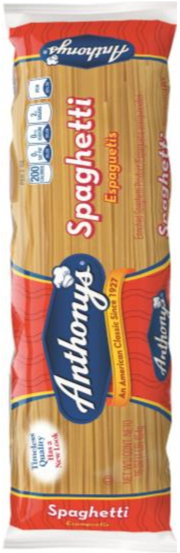

Generic Foreign USA
